# Gastrointestinal Digestion Model Assessment of Peptide Diversity and Microbial Fermentation Products of Collagen Hydrolysates

**DOI:** 10.3390/nu13082720

**Published:** 2021-08-07

**Authors:** Christina E. Larder, Michèle M. Iskandar, Stan Kubow

**Affiliations:** School of Human Nutrition, McGill University, 21111 Lakeshore, Ste-Anne-de-Bellevue, QC H9X3V9, Canada; christina.larder@mail.mcgill.ca (C.E.L.); michele.iskandar@mcgill.ca (M.M.I.)

**Keywords:** nutraceutical, in vitro digestion, collagen hydrolysate, short-chain fatty acids, branched-chain fatty acids, osteoarthritis, ammonium, hydrogen sulfide, antioxidant, peptide sequencing

## Abstract

Osteoarthritis (OA), the most common form of arthritis, is associated with metabolic diseases and gut microbiome dysbiosis. OA patients often take supplements of collagen hydrolysates (CHs) with a high peptide content. Following digestion, some peptides escape absorption to induce prebiotic effects via their colonic fermentation to generate short-chain fatty acids (SCFAs), branched-chain fatty acids (BCFAs) and colonic gases (NH_4_ and H_2_S). The capacity of CHs to generate microbial metabolites is unknown. Proteomic analysis of two CHs (CH-GL and CH-OPT) demonstrated different native peptide profiles with increased peptide diversity after in vitro gastric and small intestinal digestion. Subsequent 24 h fermentation of the CH digests in a dynamic gastrointestinal (GI) digestion model containing human fecal matter showed that CH-OPT increased (*p* < 0.05) H_2_S, SCFAs (propionic, butyric and valeric acids), BCFAs, and decreased NH_4_ in the ascending colon reactor with no major changes seen with CH-GL. No major effects were observed in the transverse and descending vessels for either CH. These findings signify that CHs can induce prebiotic effects in the ascending colon that are CH dependent. More studies are needed to determine the physiological significance of CH-derived colonic metabolites, in view of emerging evidence connecting the gut to OA and metabolic diseases.

## 1. Introduction

Osteoarthritis (OA) is the most common form of arthritis, affecting 50% of people over 75 years old, and accounting for 25% of visits to family doctors [1,2,3]. OA results in pain, mobility limitations and significant swelling in joint areas, most often in the knees and hips. Risk factors include aging, genetic predisposition, previous injuries, sex, but is also highly associated with metabolic diseases and conditions such as obesity, diabetes, hypertension and dyslipidemia [4,5,6,7,8]. The link between metabolic diseases and OA has become increasingly significant, such that the 2021 Osteoarthritis Research Society International (OARSI) Virtual World Congress held dedicated sessions on metabolic pathways and disorders contributing to OA [9]. Additionally, OA is also associated with an increased risk of metabolic syndrome [10,11]. In a comprehensive study of the National Health and Nutrition Examination Survey III cohort, results showed that the prevalence of metabolic syndrome was increased in patients with OA, regardless of age and BMI [10]. Further studies, following approximately 1000 patients over 20 years, have established that type 2 diabetes is a significant risk factor for severe OA, again independent of age and BMI [10].

The treatment options for OA are currently limited; however, several clinical trials have shown that ingestion of collagen hydrolysates (CHs) allows for decreased pain and increased mobility [12,13,14,15,16,17,18]. CH supplements contain a cocktail of peptides and amino acids (AAs); however, it is possible that these peptides are further broken down into bioactive peptides (BAPs) in the stomach and small intestine (SI) [19,20,21,22,23]. BAPs found in collagen products, such as Pro-Hyp, have been shown to decrease the loss of chondrocytes, prevent cartilage thinning, regulate genes associated with joint integrity, reduce the loss of subchondral bone as well as regulate inflammation by inhibiting cytokines such as tumor necrosis factor-α [24,25,26]. Other BAPs noted in CHs, such as Gly-Pro-Hyp, also have a variety of biological functions which include acting as an inhibitor of dipeptidylpeptidase-IV (DPP-IV), a protein linked to type 2 diabetes, as well as being involved in platelet aggregation [27,28]. Antioxidant capacity is another bioactivity of CH-derived peptides that is screened, as this could reduce reactive oxygen species damage affecting the metabolic diseases associated with OA such as type 2 diabetes [23,29,30,31,32,33]. This could be also relevant as clinical studies have shown that an increased fecal antioxidant content is associated with improved gut function and health [34].

Despite the potential impact of BAPs on human health, a recent review has highlighted the need for more detailed studies on the production of BAPs during digestive processes in view of the sparse information on this topic [35]. Previous work involving in vitro digestion of aged beef meat demonstrated generation of BAPs, although a comprehensive characterisation of the peptides generated was not performed [36]. To date, the impact of digestive processes on the breakdown of CH-derived peptides has been sparsely investigated. Hydrolysates of Alaska pollock skin collagen that underwent simulated gastrointestinal (GI) digestion showed the generation of low-molecular-mass peptides as assessed by reverse phase high performance liquid chromatography HPLC [21]. The digests were associated with increased metal-chelating activity, angiotensin-converting enzyme (ACE) and DPP IV-inhibitory activities as well as enhanced antioxidant capacity [21].

Prebiotics are dietary components that can induce beneficial changes in the growth, activity or composition of microorganisms found in the GI tract, otherwise known as the microbiota. Microbial fermentation products of prebiotics have been implicated to provide several health benefits upon the host [37]. Prebiotics have been shown to regulate inflammation, exhibit antioxidant activity as well as reduce symptoms associated with metabolic disorders such as arthritis [5,38,39,40,41]. Enzymatic hydrolysis of proteins in the SI can yield peptides that bypass intestinal absorption of the host to be fermented by colonic bacteria [42]. Consequently, it is conceivable that the rich content of peptides and AAs present in CHs leads to the generation of microbial nitrogenous fermentation products in the colon. As the definition of a prebiotic now includes fermented proteins, peptides and AAs [43], investigation into the prebiotic effects of CHs could be important for OA as gut health has been linked to joint health [5,38]. In that regard, a recent study on obesity showed a direct link between OA and the gut microbiome, and its effects on systemic inflammation [5]. Supplementation of the prebiotic oligofructose altered the GI microbiota of OA and obese mice to a more favorable and healthier microbiota, which was associated with prevention of cartilage loss and improved joint structure [4]. Therefore, further insights as to how CHs impact on gut microbial fermentation is warranted, particularly as patients are increasingly utilizing these products to mitigate the symptoms of OA [44,45].

Short-chain fatty acids (SCFAs) are well established products of fermentation of prebiotics and their production is an indicator of a healthy microbial community [46]. SCFA assessment includes acetic, propionic and butyric acids, which are normally present in ratios ranging from 3:1:1 to 10:2:1 [40]. SCFA production is considered one of the major benefits associated with prebiotics and the relative abundance of fecal SCFAs has been used as a biomarker of gut health as well as overall systemic health [47,48]. Although only a small fraction of SCFAs is absorbed, there are numerous biological functions attributed to SCFAs that are under active investigation. For example, butyric acid has been implicated in the control of inflammation [49], appetite [50] and liver mitochondrial function [51]. Although less is known about minor SCFAs such as valeric and caproic acids, they also have the potential to affect human health [52,53].

CH supplementation could also lead to increased microbial production of branched-chain fatty acids (BCFAs; isobutyric, isovaleric, isocaproic acids), which are products derived from colonic microbial fermentation of branched-chain AAs. The health impact of BCFAs is still under debate. Increased production of BCFAs has been associated with preventing irritable bowel syndrome [54] whereas other studies have increasingly linked exposure to BCFAs with insulin resistance and obesity [55]. Other biomarkers of large intestinal GI health include ammonium (NH_4_) and hydrogen sulfide (H_2_S), which are often attributed to an over abundant quantity of proteins and some AAs available for fermentation, which can promote dysbiosis [46,56,57]. Increased production of these gases in the GI tract can adversely affect human health [46], although recent reports have indicated that low levels of H_2_S may help to avoid GI damage associated with taking nonsteroidal anti-inflammatory drugs (NSAIDS) [58].

For discovery-related investigations pertaining to nutrient and microbial metabolite assessment, human trials are limiting and impractical [59,60]. Furthermore, animal studies, often using rodents, are generally slow, costly and predictions of digestion and microbiota changes do not always align with human clinical data due to species differences in nutrient utilization, metabolic activity and host microbiota [61,62,63]. As an alternative, dynamic in vitro GI models can closely mimic human upper intestinal digestion and recreate the colonic environment similar to human in vivo conditions [59,60]. Accordingly, such models are increasingly being utilized to predict peptide digestibility and microbiome analysis [35,64], and assessment of SCFAs, BCFAs and colonic gases that provide information on the functional activity and compositional profiles of the gut microbiota [47,48,56]. As CHs continue to be widely available for OA patients, our study was designed to address the significant gaps in the literature concerning the digestibility of CHs and their potential prebiotic effects, which could impact human health. To determine the peptide profile of two commercially available CH products, upper intestinal digestion followed by proteomics analysis was completed. To observe the production of colonic microbial metabolites after CH digestion and fermentation, a dynamic multistage computer-controlled GI model was used to determine the SCFA, BCFA, NH_4_ and H_2_S content as well as changes in antioxidant capacity.

## 2. Materials and Methods

### 2.1. Upper Intestinal in Vitro Digestion of Collagen Hydrolysates

The two bovine-sourced CH products used for this study were Original Formula (Genacol, Blainville, QC, Canada) (CH-GL) and Selection (Uniprix, Saint-Léonard, QC, Canada) (CH-OPT). Upper intestinal digestion involving the stomach and SI was adapted from Alemán et al., 2013 and Miranda et al., 2013 [20,65]. CHs (1200 mg) were digested in reactor vessels placed in a Versa Water Bath at 37 °C (Fisher Scientific, model 224, Waltham, MA, USA), with continuous stirring and the pH was monitored and adjusted throughout digestion (Fisher Scientific, S90528, Waltham, MA, USA). Exactly 1 mL of an enzyme solution of α-amylase (0.70783 g in 1.5 mL ddH_2_O; Sigma-Aldrich, A3176, St. Louis, MO, USA) was added to each vessel and incubated for 15 min at a pH of 6.9. A pepsin solution (1.167 g; Sigma-Aldrich, P7125, St. Louis, MO, USA) was prepared in 0.1 M HCL, of which 2 mL was added and the pH was adjusted to 2. The vessels were incubated for 30 min. Following this, 2 mL of a bile solution (0.9 g/L pancreatin (Sigma-Aldrich, P7545, St. Louis, MO, USA), 6 g/L bile extract (Sigma-Aldrich, B8631, St. Louis, MO, USA), and 12 g/L sodium bicarbonate) prepared in ddH_2_O was added. The pH was adjusted to 8 and the solution incubated for 120 min. The digesta was then rapidly cooled on ice and frozen to stop the enzymatic processes. Subsamples of the digesta were filtered using a 0.45 µm Millipore syringe-driven filter and stored at −20 °C until analysis.

### 2.2. Peptide Profile

#### 2.2.1. Matrix Assisted Laser Desorption/Ionization (MALDI)

Upper intestinal digesta were processed using Matrix Assisted Laser Desorption/Ionization Time-of-Flight (MALDI-TOF). Samples were centrifuged for 10 min to eliminate floating particles and then ZipTiped (ThermoFisher Scientific 87782, Waltham, MA, USA), as per the manufacturer’s instructions. From the ZipTiped samples, 1 μL was placed onto a MALDI target (MTP 384 target ground steel BC) and left to fully air dry. Once dried, 1 μL of matrix (10 mg/mL α-cyano-4-hydroxycinnamic acid, with 1:1 acetonitrile and 0.1% trifluoroacetic acid) was added on top of the sample and again left to air dry completely. The loaded plates were then inserted in the MALDI-TOF/TOF instrument. Profiling was performed on a MALDI-TOF/TOF Ultraflextreme mass spectrometer equipped with a SmartBeam II Nd:YAG 355 nm laser operating at 2000 Hz (Bruker Daltonics, Billerica, MA, USA). MS data were acquired by accumulating 1500 laser shots per spot in a mass range of 300–4000 Da. External calibration was carried out using a homemade standard peptide mix. Data analysis was performed with FlexAnalysis 3.4 (Bruker Daltonics).

#### 2.2.2. Proteomic Analysis

Both CH samples before and after upper intestinal digestion were assessed for peptide diversity. Samples were reduced and alkylated with dithiothreitol (Sigma-Aldrich, 10197777001, St. Louis, MO, USA) and iodoacetic acid (Sigma-Aldrich, I4386, St. Louis, MO, USA) respectively, then digested with mass spectrometry (MS)-grade trypsin (Thermo Fisher Scientific 90057, Waltham, MA, USA). Samples in 2% acetonitrile, 98% water, 0.1% formic acid were loaded onto a Thermo Acclaim Pepmap precolumn (Thermo, 75 µM ID X 2 cm, C18, 3 µM beads) and then onto an Acclaim Pepmap Easyspray analytical column separation (Thermo, 75 µM × 15 cm, C18, 2 µM beads) using a Dionex Ultimate 3000 uHPLC at 220 nL/min with a 120 min analytical gradient of 2–35% organic solvent (0.1% formic acid in acetonitrile). The column was flushed using 80% organic solvent for 20 min before re-equilibrating back to 2% organic solvent for 20 min. Blank solvent was injected in between samples and the column was then flushed for 60 min at 80% organic solvent and equilibrated with 2% organic solvent for 20 min. Peptides were sequenced using a Thermo Orbitrap Fusion mass spectrometer (120,000 FWHM resolution at 200 amu in MS1; mass range 375–2000, sprayer voltage +1850V). MS/MS sequencing was performed using higher-energy collisional dissociation (HCD) fragmentation (30%; 15,000 resolution, 1.8 amu wide quadrupole isolation) at top speed for all peptides with a charge of 2+ or greater using a cycle time of 3 s before the next MS1. An MS/MS exclude time of 12 s was used. Peptide data was searched and compared using the Mascot 2.3 search engine (Matrix Science, Boston, MA, USA) against bovine sequences (Uniprot), corresponding to the source of the materials. Database search results were loaded onto Scaffold Q+ Scaffold_4.4.8 (Proteome Sciences, Addlestone, Surrey, UK) for analysis. Peptide sequences determined were from 300 to 4000 *m*/*z*. BIOPEP-UWM database was used to search for BAP sequences [66].

### 2.3. Dynamic In Vitro Gastrointestinal Digestion of Collagen Hydrolysates

An established dynamic computer-controlled GI model was used to digest the CH products, which has been previously validated [67,68]. The model consists of five bioreactor vessels: stomach, SI, ascending colon, transverse colon and descending colon. For each vessel, the pH was continuously measured and adjusted by a computer system, with either the addition of 0.2 M NaOH or 0.5 M HCl. The temperature of the GI model was kept at 37 °C and was monitored and controlled by flowing water through double-jacketed reactor vessels in which the GI bioreactor components are found. The model components are attached by plastic tubing and the contents of the reactor vessels were moved by peristaltic pumps. The vessel contents were continuously agitated using magnetic stir plates. The colonic vessels were inoculated with fecal matter and allowed to stabilize over a two-week period to allow for optimal bacterial growth. The two bovine-sourced CH products (CH-GL and CH-OPT) used for upper intestinal digestion (Section 2.1) were again used in the dynamic digestion model. A CH treatment dose of 1200 mg was added to a GI food mixture, as previously described by Ekbatan et al., (2016) [68] and Gaisawat et al., (2019) [69], and which was slowly pumped into the stomach vessel. The treatment dose was based on the daily dose of the Genacol Original Formula^®^ that was shown to reduce joint pain in clinical trials [12,13,18] and the same dose was used for the upper intestinal digestions (see Section 2.1). To our knowledge, no information is available regarding the clinical efficacy of the Selection CH product. An enzyme solution of α-amylase (Sigma-Aldrich, A3176, St. Louis, MO, USA) prepared in sterile deionized water was added to the GI food mixture to replicate salivary digestion. Pepsin (Sigma-Aldrich, P7125, St. Louis, MO, USA) prepared in 0.1 M HCl was added to the stomach vessel and 35 mL of a bile solution composed of pancreatin (Sigma-Aldrich, P7545, St. Louis, MO, USA), bile extract (Sigma-Aldrich, B8631, St. Louis, MO, USA) and sodium bicarbonate were added to the SI, as described by Ekbatan et al., (2016) and Gumienna et al., (2011) [68,70]. Sub-samples from each vessel were obtained at times 0, 8, 16 and 24 h and filtered using a 0.45 µm Millipore syringe-driven filter. Two separate digestion runs were completed for each treatment, with a washout/re-stabilization period of 3 days between treatments. Previous in vitro fermentation experiments have also utilized two separate digestion runs [71].

### 2.4. Colonic Gases

#### 2.4.1. Ammonium (NH_4_) Content

The following method was adapted from Gaisawat et al., (2019) [72]. A 1000 ppm stock solution of NH_4_ (Sigma-Aldrich, A4418, St. Louis, MO, USA) was prepared in water, along with subsequent dilutions for the standard curve. Samples (50 µL) or standards were pipetted into a 96-microplate well in triplicate. To each well, 25 µL of a citrate reagent, 25 µL of freshly prepared hypochlorite reagent and 145 µL of deionized water were added. The citrate reagent comprised of 5 g trisodium citrate (Sigma-Aldrich, 1110371000, St. Louis, MO, USA) with 2 g of NaOH in 100 mL deionized water with 30 µL of salicylate nitroprusside reagent (7.813 g sodium salicylate (Sigma-Aldrich, S3007, St. Louis, MO, USA) with 0.125 g sodium nitroprusside (Sigma-Aldrich, 1614501, St. Louis, MO, USA) in 100 mL of deionized water, and adjusted to pH 6.5). The hypochlorite reagent was made with 1 g Na_3_PO_4_ with 2 mL 2 M NaOH, 10 mL Javex bleach in 100 mL deionized water and pH adjusted between 12 and 13. The microplate was covered, gently rocked back and forth, and allowed to sit at room temperature (RT) for 30 min. The absorbance of the samples and standards was read using a microplate reader (µQuant, 140084, BioTek Instruments, Winooski, VT, USA) at 650 nm. The concentrations of the samples were calculated using an external calibration curve, where linearity was assessed using R^2^.

#### 2.4.2. Hydrogen Sulfide (H_2_S) Content

The following spectrophotometric method was used to determine inorganic sulfide concentration and was adapted from Gaisawat et al., (2019) [72]. A standard stock solution of 0.1 mM sodium sulfide in oxygen free water was prepared, along with subsequent dilutions for the standard curve. Solution A was prepared using a 5:1 ratio of zinc acetate (2.5% in water) to NaOH (6% in water). Inside a 15 mL centrifuge tube, 0.5 mL of Solution A was added to 0.3 mL of digesta. The tubes were shaken and centrifuged at 3000 *g* for 10 min. The supernatant was carefully decanted, and the pellet washed once with 5 mL of 1.5 M NaCl (pH 8), and then with 5 mL of distilled water (pH 8). The pellet was resuspended in 0.7 mL of water and vortexed. To each sample, 0.25 mL of 0.1% N,N-dimethyl-p-phenylenediamine monohydrochloride (Sigma-Aldrich, D5004, St. Louis, MO, USA) in 5.5 N HCL was added and shaken. Exactly 0.1 mL of 1.15 mM ferric chloride (Sigma-Aldrich, 157740, St. Louis, MO, USA) in 0.6 N HCl was added. A volume of 200 µL of each sample and standard was pipetted into a 96-well microplate in duplicate and allowed to incubate for 30 min at RT. The absorbance of the samples and standards was read at 650 nm using a microplate reader (µQuant, Bio-tek Instruments, model: 140084, Winooski, VT, USA). The concentration of the samples was calculated using an external calibration curve, where linearity was assessed using R^2^.

### 2.5. Short- and Branched-Chain Fatty Acids

The SCFA and BCFA content was measured using a gas chromatograph system equipped with a flame ionization detector (GC-FID) (6890A series, Agilent Technologies, Santa Clara, CA, USA) using an adapted method from Ekbatan et al., (2016) and Gaisawat et al., (2019) [68,69]. Pre-filtered subsamples from the GI model for each colonic vessel were obtained, and then diluted 1:1 with methanol. A 1 µL volume was injected into the GC-FID system. An HP-INNOWAS 30 m fused capillary column (Agilent Technologies, Santa Clara, CA, USA) with a 250 µm ID and a film thickness of 0.25 µm was used to separate the SCFAs and BCFAs from both the standard mixes and samples. A flow rate of 1 mL/min of helium gas was used. The inlet and detector temperatures were set at 220 °C and 230 °C, respectively. The oven temperature was originally set at 150 °C and held for 10 min and then increased by 10 °C/min to 180 °C and held for 5 min. SCFAs and BCFAs were identified based on retention times using a standard mix (Sigma-Aldrich, 46975-U, St. Louis, MO, USA) and quantified (mM) using an external calibration curve, based on peak area and dilutions of the standard mix (Figure S1). Linearity of SCFA and BCFA calibration curve was assessed using R^2^; all were above 0.99.

### 2.6. Antioxidant Capacity

#### 2.6.1. Ferric Reducing Ability of Plasma (FRAP) Assay

The following method was adapted from Gaisawat et al., (2019) [69] and Benzie and Strain (1996) [73]. A 1 mM stock solution of ascorbic acid (Sigma-Aldrich, A7506, St. Louis, MO, USA) was made and subsequent dilutions completed to obtain a standard curve. A 96-well microplate was used, where 10 µL of either sample or standard was pipetted into a well, along with 30 µL of deionized and 200 µL of a previously made FRAP solution (acetate buffer, 2,4,6-tri(2-pyridyl)-s-triazine and ferric chloride solution combined in a ratio of 10:1:1). The samples and standards were mixed by pipetting for 10 s and then incubated at RT for 8 min. The absorbance was measured at 593 nm using a µQuant microplate reader (BioTek Instruments, Winooski, VT, USA). The antioxidant capacity of the samples was calculated using an external calibration curve, where the linearity of the curve was assessed using R^2^.

#### 2.6.2. DPPH (2,2-Diphenyl-1-picrylhydrazyl) Assay

The method was adapted from Gaisawat et al., (2019) [69] and is based on the reduction of the free radical 2,2-diphenyl-1-picrylhydrazyl (DPPH). A standard curve was made from a 50 mM Trolox stock solution, with subsequent dilutions using methanol. A 1 mM stock DPPH solution was diluted with methanol to obtain an absorbance between 0.9–0.5 to form a working solution. Exactly 100 µL of gut digesta sample or standard was pipetted into a 96 well plate, along with 150 µL of DPPH working solution and left to incubate at room temperature for 30 min in the dark. Absorbance was measured at 517 nm using a µQuant microplate reader (BioTek Instruments, Winooski, VT, USA). The antioxidant capacity of the samples was calculated using an external calibration curve, where the linearity of the curve was assessed using R^2^.

### 2.7. Statistical Analysis

Data is reported as mean ± standard error of the mean (SEM). For each treatment, differences between timepoints were assessed using a one-way ANOVA, followed by Dunnett’s post hoc test, using time 0 h as control. All analyses were completed using JMP Pro (JMP^®^, Version 15.1.0, 2019 SAS Institute Inc., Cary, NC, USA) and results were considered statistically significant if *p* < 0.05. Figures were made using GraphPad Prism (Version 9.0.1 for Windows, GraphPad Software, San Diego, CA, USA.)

## 3. Results

### 3.1. Peptide Profile

Before digestion, CH-GL had 62 peptide sequences not shared with CH-OPT, whereas CH-OPT had 17 peptide sequences not found in CH-GL (Table S1). Additionally, 3 peptide sequences were shared between the two types of CH brands. After upper intestinal digestion, both CH products had an increase in peptide diversity (Table S2). CH-GL had 300 peptide sequences not found in CH-OPT after digestion, whereas CH-OPT had 574 sequences not observed in CH-GL. After digestion, 138 peptide sequences were shared between CH-GL and CH-OPT.

Sequences released after digestion were searched using the BIOPEP-UWM^TM^ database to determine the bioactivity of the peptides metabolized; no bioactive peptide sequences were found, regardless of CH treatment. However, some sequences known to be bioactive registered in BIOPEP were found within the peptides, and often at the c-terminus of the peptide sequences, which could be released upon further digestion. Specifically, the BAPs PR and PQ which have ACE-inhibitory activity, and GPV which shows ACE-inhibitory activity as well as DPPIV inhibitory activity were found at the c-terminus of multiple peptides post-digestion (Table S2) [36,60].

The general peptide profile of both CH products was also determined using MALDI. Although from the same collagen source, the peptide profile, distribution, and content of both CH products were different both before and after digestion (Figures S2–S5). After both CH products were digested in the stomach and SI, an increase in peptide peaks was observed. The general peptide profile and intensity of the peptide peaks were different between CH-GL and CH-OPT for low and higher molecular weight peptides after digestion (Figures S4 and S5).

### 3.2. Colonic Gases

**Figure 1:**
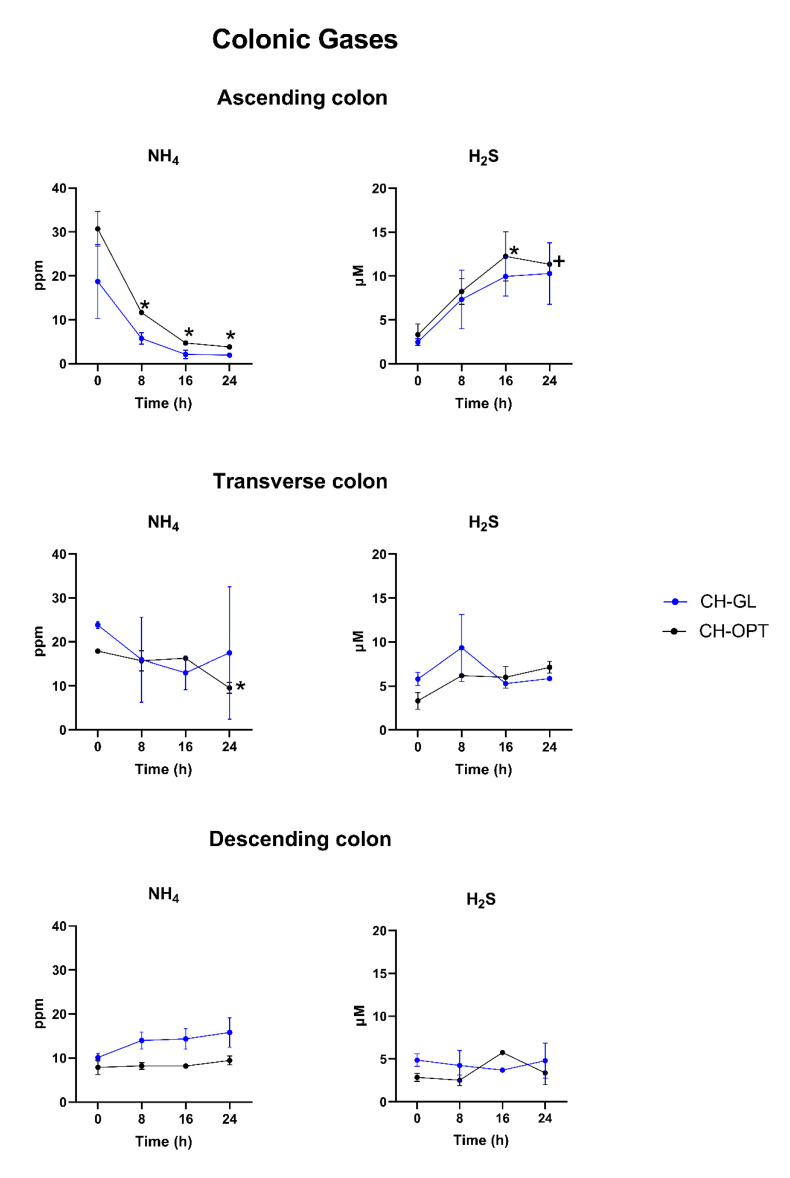
NH_4_ and H_2_S content for CH-GL and CH-OPT over time for each colonic region. Values are expressed as mean ± SEM in ppm for NH_4_ and µM for H_2_S. The * symbol indicates a significant difference from control (time 0 h) (*p* < 0.05) for each treatment and colonic region. The symbol + indicates a possible trend (*p* = 0.0654).

For each colonic vessel, no significant differences in NH_4_ and H_2_S were observed between baseline control (time 0 h) and each timepoint (8, 16, 24 h) after the digestion of CH-GL (Figure 1). In the ascending colon, a significant decrease (*p* < 0.05) in NH_4_ (ppm) content was observed after 8, 16 and 24 h (11.64 ± 0.25, 4.71 ± 0.35, 3.81 ± 0.11, respectively) following CH-OPT supplementation compared to 30.71 ± 3.92 at baseline (time 0 h) (Figure 1). In the transverse colon, a decrease in NH_4_ (ppm) content was also observed after CH-OPT supplementation but only after 24 h of digestion (9.55 ± 1.24), and no difference in NH_4_ content was observed in the descending colon. There were no significant changes in H_2_S (µM) content after digestion of CH-OPT for each timepoint and colonic vessel except for an increase in H_2_S (µM) content in the ascending colon from 3.333 ± 1.238 at baseline (time 0 h) to 12.238 ± 2.810 after 16 h (*p* < 0.05).

### 3.3. SCFAs and BCFAs

In the ascending colon, no changes in SCFAs were observed after CH-GL digestion (Table 1) whereas individual SCFA profiles showed variability between baseline control (time 0 h) and after the digestion of CH-OPT (Table 2). Specifically, propionic acid (mM) content increased from a baseline value of 0.50 ± 0.47 to 7.59 ± 0.59 and 6.53 ± 1.71 after 16 h and 24 h, respectively. Similarly, a significant (*p* < 0.05) increase in butyric acid (mM) was also observed after 16 and 24 h (6.97 ± 0.20 and 5.78 ± 1.21, respectively) from time 0 (2.92 ± 0.21). Valeric acid also increased after 8, 16 and 24 h after CH-OPT fermentation. No significant changes in acetic acid were reported for CH-OPT, for any timepoint. No changes in caproic acid or heptanoic acid were observed in the ascending colon for either CH treatment. Furthermore, for both CHs, no changes in SCFA or BCFA content were observed in the transverse and descending colon compared to baseline (time 0 h).

No increase in BCFA content was observed after digestion of CH-GL, although a significant decrease in isobutyric acid (mM) was detected in the ascending colonic reactor (Table 1). Isobutyric acid (mM) decreased from 0.40 ± 0.05 at baseline to 0.19 ± 0.01 after 24 h (*p* < 0.05). A trend for isobutyric acid to decrease relative to control time 0 h was also observed at 8 h (0.23 ± 0.04) (*p* = 0.0531).

An increase in BCFAs in the ascending colonic reactors was observed only with CH-OPT supplementation (Table 2). Specifically, isovaleric acid (mM) increased from 2.20 ± 0.09 at baseline to 3.69 ± 0.34 after 16 h (*p* < 0.05). Although not significant, a trend for an increase in isovaleric acid was observed after 24 h (*p* = 0.0588).

Similarly, as observed for SCFAs, there were no changes in BCFAs in the transverse and descending colon vessels for either CH treatment.

### 3.4. Antioxidant Capacity

**Figure 2:**
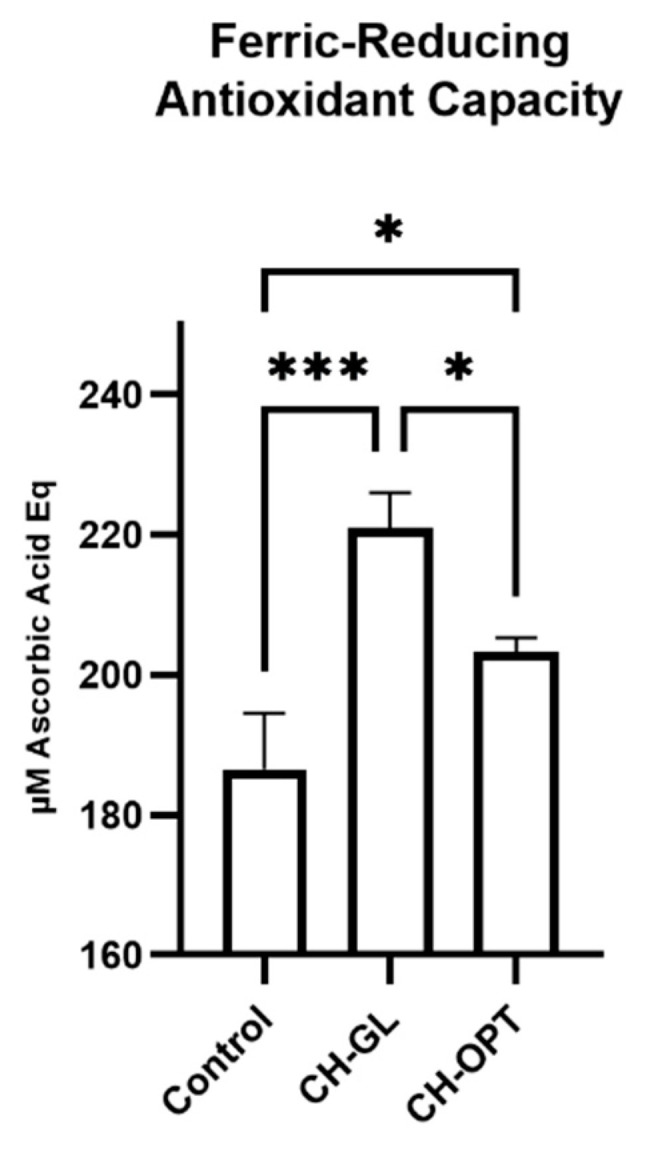
Ferric-reducing antioxidant capacity of CH-GL and CH-OPT after upper intestinal digestion. Values are expressed as mean ± SEM in µM ascorbic acid Equation. One-way ANOVA followed by Tukey-HSD was completed where *p* < 0.05 was considered significant. Columns with asterisks are significantly different (* *p* < 0.05, *** *p* < 0.001).

After upper intestinal digestion, there was a significant increase in ferric-reducing antioxidant capacity (FRAP) between a control digestion (with no CH supplementation), CH-GL and CH-OPT (Figure 2). Both CH treatments were significantly greater in antioxidant capacity compared to control. Furthermore, the antioxidant capacity of CH-GL in the SI was greater than that of CH-OPT.

There were no significant differences in antioxidant capacity (DPPH and FRAP) after CH-GL supplementation at any timepoint (8, 16, 24 h) for the ascending, transverse and descending colonic vessels (Table S3). Conversely, after CH-OPT supplementation, a significant increase (*p* < 0.05) in DPPH radical scavenging activity (mM Trolox Eq) from baseline (17.53 ± 0.68) was seen after 16 and 24 h of digestion (28.25 ± 0.85 and 26.88 ± 1.28, respectively), although only in the ascending colon (Table S3). No changes in DPPH capacity were seen in the transverse and descending colon. Furthermore, no changes in FRAP were seen after CH-OPT supplementation.

## 4. Discussion

This work addressed significant gaps in the literature concerning the upper intestinal digestibility of bovine CHs and well as their potential prebiotic effects at the level of the colon. Differences in the peptide profiles before and after upper intestinal digestion between the two CH products were observed, as supported through MALDI and proteomics analyses. Before digestion, three peptide sequences were shared between the two CHs, whereas 62 sequences were only found in CH-GL, and 17 sequences seen only in CH-OPT. Although the CH products shared 138 peptide sequences after digestion, peptidomic results characterized the vast heterogeneity of peptide sequences generated after CH-OPT and CH-GL digestion as exemplified by MALDI profiles as well as 300 peptides being found only in CH-GL and 574 peptide sequences noted solely in CH-OPT. The difference in peptide diversity can result from differing collagen hydrolysate preparation or purification methods as well as upper intestinal digestion [23,31,32]. The contrasting peptide profiles seen post-digestion between the two supplements could provide partial explanation as to why the antioxidant capacity of CH-GL was greater after upper intestinal digestion compared to CH-OPT. Previous studies have indicated that digestion of tuna skin collagen hydrolysates leads to an increase in antioxidant capacity, which was associated with lower molecular weight peptides [74]. Although the peptide sequences released after digestion did not match any peptides from the database BIOPEP-UMW, this was the first study to characterize peptides before and after digestion of bovine sourced CHs. Furthermore, known BAPs such as PR, PQ, and GPV from collagen were found within the peptides sequenced in both CH products, often at the c-terminals. It is conceivable that further metabolism could occur in the colonic regions, easily cleaving c-terminal amino acids, thereby releasing these BAPs. Verification of further proteolytic metabolism in the colon remains to be tested. Future use of dynamic gastrointestinal models could provide a platform to investigate the release of BAPs after colonic metabolism and the potential physiological significance of the BAPs. It is also important to note that novel research into identifying bioactive peptides is still ongoing and current databases are continuously updated. Thus, although no sequences post-digestion were identified as being bioactive, future research might establish bioactivities for some of those sequences.

Using the in vitro dynamic GI model, new insights were obtained in terms of the production of microbial metabolites generated via fermentation of the SI digestion end-products of hydrolyzed collagen by human gut microbiota. Dynamic GI models, such as the one used herein, allow for multiple and simultaneous sampling from each colonic region, which is not possible to perform with in vivo studies due to ethical and accessibility issues. Dynamic GI models provide a platform for higher throughput analysis of the post-digestive end-products of nutrients, food components and their microbial metabolites. These models provide an alternative to costly and potentially non-representative animal studies, particularly as differences in metabolism and host microbiota can often affect results. Such GI models have certain limitations, such as variability of fecal matter used to inoculate the colonic vessels that can lead to differences in host microbiota composition and metabolism. Additionally, these models do not provide information of the crosstalk between gut microbiota and intestinal cells, which affects host inflammatory pathways and the innate immune system.

Although both hydrolysates were derived from bovine collagen, only the CH-OPT treatment was associated with an increase in colonic SCFA and BCFA content. Furthermore, only CH-OPT showed an increase in H_2_S and antioxidant capacity with a corresponding decrease in NH_4_, although those outcomes were primarily seen in the ascending colonic region. These findings are most likely due to differences in the SI peptide profiles between the two CH products as discussed above. In support of this contention, greater amounts of peptide sequences larger then 6 AA residues totalling 574 in CH-OPT versus 300 in CH-GL, remained intact following upper GI digestive processes to promote changes in antioxidant capacity in the ascending region of the colon and induce microbial generation of SCFAs in terms of butyric, propionic and valeric acids and the BCFA, isovaleric acid. As no changes in SCFAs, BCFAs, H_2_S and antioxidant capacity were observed in the transverse or descending colonic vessels for either CH, it is likely that insufficient amounts of peptides reached those vessels to support further microbial fermentation and changes to the microbiota. Studies investigating the bioavailability of CHs are needed, to verify if peptides from CH-GL formed during digestion are absorbed locally at the GI tract and survive after they permeate across the intestinal epithelium to enter the systemic blood circulation. Furthermore, investigations focusing on lower MW CH peptides are needed, as di- and tri-peptides from collagen have known bioactivity, and increased bioavailability compared to greater MW CH peptides [19,22,35]. Analysis identifying lower MW peptides continues to be a limitation of “peptide-centric” proteomic work, seeing as di- and tri-peptides are too small for sequencing. These small MW peptides only generate 1+ ions, and the signal interference from other ions coming from solvents, plasticizers, silicates, etc., overwhelm the peptide response. Larger MW peptides (15 AA+) provide stronger signals, with mainly 2+ ions and background ionic noise does not interfere. For this reason, many peptide sequencing approaches mainly focus on higher MW peptides. Methodologies adapted from urine samples using liquid chromatography-MS and capillary electrophoresis-MS/MS could provide novel approaches to detect lower MW peptide from simulated GI digestion, but require further development and verification [75]. However, current efforts by us to assess for lower MW BAPs, such as the di- and tri-peptides Pro-Hyp and Gly-Pro-Hyp, are ongoing and preliminary methodology results using capillary electrophoresis are encouraging [76].

Although there are no analogous studies involving CH fermentation, an increase in butyrate and propionate content was observed from fermentation of casein hydrolysates using single stage, anaerobic fermentation chambers inoculated with human fecal matter [77]. In contrast to the present work, the latter study did not include stomach and SI digestive processes that can modify peptide profiles prior to their exposure to microbial metabolism. Other reports have shown that wheat arabinogalactan peptides were associated with an increase in SCFAs after 24 h, although this was assessed using in vitro batch fermentation rather than a dynamic GI model system.

There are possible metabolic health benefits that might accrue from increased colonic generation of propionic and butyric acids that was associated with CH-OPT supplementation [47,48], and a decrease in NH_4_ content [46,56], seeing as when NH_4_ levels are greater then 5–10 mM, this can have negative health consequences by altering the metabolism of intestinal cells, impairing DNA synthesis and reducing the life expectancy of cells [46]. These changes encourage the multiplication of damaged cells in the intestine with altered metabolism. Levels of NH_4_ reported in this paper are closer to the lower levels reported in the literature [46], and were decreased further after CH-OPT supplementation whereas no change was reported with CH-GL. Besides NH_4_ in the colon, dysbiosis is also observed with a high production of H_2_S content, which is another microbial biomarker of the large intestine and associated with high levels of fermented protein and sulfur containing amino acids [46,56]. Levels of H_2_S measured after CH supplementation were much lower than levels shown to cause significant DNA damage (250 µM) [46]. Furthermore, H_2_S at low concentrations has recently been reported to be a beneficial gas produced in the GI tract, by helping to prevent dysbiosis and avoid GI damage associated with taking NSAIDS [58].

The benefits of SCFA production and improvements to GI gas content seen with the CH-OPT treatment could be partially offset by the corresponding increase in isovaleric acid, since enhanced gut exposure to BCFAs has been linked to an increased risk for diabetes and obesity [55]. Furthermore, although not much information is currently known about the health modulatory properties of minor SCFAs, recent research has suggested that fecal valeric acid may serve as an indicator of gut microbial dysbiosis [52]. Hence, the increase in valeric acid concentrations observed with CH-OPT could be indicative of adverse changes in gut microbial composition. An additional potential concern are reports that fecal valeric acid is positively correlated with the pro-inflammatory C-reactive protein in patients with ischemic stroke [78]. Conversely, the conjugated base of valeric acid has been associated with enhancing interleukin-10 production and suppressing Th17 cells, which could provide anti-inflammatory benefits [79]. The immunomodulatory effects of valeric acid need further investigation, particularly in relation to OA and rheumatoid arthritis as these are conditions associated with an increase in joint and whole body proinflammatory processes [80]. Interestingly, the lack of effect of the CH-GL on the SCFA and BCFA production and other microbial biomarkers of NH_4_ and H_2_S indicates that this supplement has neither prebiotic nor dysbiotic properties in contrast to CH-OPT.

As CH supplements continue to grow in popularity and are widely available for OA patients, our study was designed to address the significant literature gaps concerning the digestibility of CHs and their potential prebiotic effects. The effects of microbial metabolite production after CH supplementation may not only depend on the CH product fermented, but also on the initial dose of supplement. The treatment dose used in this study was based on the daily dose of the Genacol Original Formula^®^ that was shown to reduce joint pain in clinical trials [12,13,18]. Other clinical studies, however, have used much greater doses ranging from 5 to 35 g of hydrolyzed collagen products [14,15,17,19,81,82]. It is conceivable that with a higher initial dose of CHs, greater microbial fermentation could have occurred due to more substrate availability for fermentation with subsequent greater increases in SCFAs, BCFAs, colonic gas production and antioxidant capacity. The effective dose regarding pain management but also colonic metabolite production needs to be further investigated, as there is currently no standardized treatment dose. Our work is the first to establish that CH products utilized by OA patients can exert prebiotic effects, particularly in the ascending colon. Further research is needed using 16S rRNA gene amplicon sequencing to profile gut microbiota community structure and composition as affected by CH supplementation. It is possible that an increase in beneficial colonic metabolites could improve joint structure as well as prevent cartilage loss, as recent research has suggested a connection of the gut microbiome to OA [4]. Supplementation using the prebiotic oligofructose to obese OA mice changed the host microbiota to a healthier profile, notably by supporting the growth of *Bifidobacterium pseudolongum*. Beneficial changes to the microbiome were associated with decreased systemic inflammation, which decreased OA progression by regulating joint inflammation, chondrocyte hypertrophy, osteophyte formation, as well as joint mineralization.

## 5. Conclusions

To date, there is limited information regarding the digestion of food-derived peptides and the effects on the gut microbiome and microbial fermentation products such as SCFAs, BCFAs, NH_4_ and H_2_S. The present study provides the first evidence and characterization of peptides released after upper intestinal digestion. Furthermore, this study also provides first evidence that CHs can lead to the generation of SCFAs and BCFAs, although this microbial metabolic activity appears to be dependent on the nature of the CH tested, which corresponds to differing peptide diversities after upper intestinal digestion. Interestingly, changes to biomarkers of microbial health primarily only affect the ascending colon, indicating that CH products provide insufficient peptide and AA material to the transverse and descending colon. A recent review has highlighted that, long-term dietary choices such as greater protein content could exert effects on GI microbial populations, which has implications towards development of metabolic diseases such as obesity and diabetes [43]. This review emphasized that important knowledge gaps exist concerning dietary protein-mediated generation of colonic microbial molecules that could exert bioactivities towards gut inflammation and permeability. Accordingly, it is possible that CH supplements, which have a rich peptide content, can impact the structure and function of gut microbial communities. Dynamic GI model platforms, such the one utilized in the present study, can be a useful tool to further investigate the impact of CH supplementation on the gut microbiota to more fully understand the impact of these nutraceuticals on GI and systemic health.

## Figures and Tables

**Table 1:** SCFA and BCFA for CH-GL at times 0, 8, 16 and 24 h for each colonic region.

	SCFA						BCFA		
Time (h)	Acetic Acid	Propionic Acid	Butyric Acid	Valeric Acid	Caproic Acid	Heptanoic Acid	Isobutyric Acid	Isovaleric Acid	Isocaproic Acid
	Ascending colon								
0	12.43 ± 4.43	0.08 ± 0.07	0.05 ± 0.02	0.00 ± 0.00	0.00 ± 0.00	0.00 ± 0.00	0.40 ± 0.05	0.04 ± 0.01	0.00 ± 0.00
8	24.83 ± 1.84	0.16 ± 0.05	0.05 ± 0.02	0.00 ± 0.00	0.00 ± 0.00	0.00 ± 0.00	0.23 ± 0.04 ^+^	0.04 ± 0.00	0.00 ± 0.00
16	25.17 ± 1.95	0.18 ± 0.04	0.05 ± 0.00	0.00 ± 0.00	0.00 ± 0.00	0.00 ± 0.00	0.26 ± 0.01	0.04 ± 0.00	0.01 ± 0.01
24	21.48 ± 0.20	0.22 ± 0.06	0.05 ± 0.00	0.00 ± 0.00	0.01 ± 0.01	0.00 ± 0.00	0.19 ± 0.01 *	0.03 ± 0.01	0.01 ± 0.00
	Transverse colon								
0	3.10 ± 0.67	1.34 ± 1.27	4.67 ± 3.21	1.56 ± 1.24	0.43 ± 0.43	0.00 ± 0.00	0.54 ± 0.17	0.35 ± 0.18	0.01 ± 0.01
8	6.87 ± 2.68	1.52 ± 1.49	5.26 ± 2.65	1.74 ± 1.12	0.41 ± 0.38	0.00 ± 0.00	0.55 ± 0.09	0.43 ± 0.22	0.01 ± 0.01
16	10.56 ± 6.86	1.74 ± 1.72	3.85 ± 2.35	1.42 ± 1.09	0.29 ± 0.03	0.00 ± 0.00	0.40 ± 0.23	0.35 ± 0.27	0.02 ± 0.01
24	12.2 ± 10.56	1.29 ± 0.51	2.16 ± 1.14	0.85 ± 0.54	0.14 ± 0.12	0.00 ± 0.00	0.32 ± 0.10	0.26 ± 0.16	0.02 ± 0.01
	Descending colon								
0	4.50 ± 1.73	2.08 ± 1.90	4.56 ± 3.54	1.09 ± 1.60	0.46 ± 0.42	0.00 ± 0.00	0.79 ± 0.33	0.50 ± 0.26	0.01 ± 0.01
8	5.56 ± 1.12	1.75 ± 1.51	4.70 ± 1.98	1.80 ± 0.99	0.41 ± 0.32	0.00 ± 0.00	1.00 ± 0.53	0.60 ± 0.11	0.01 ± 0.01
16	3.83 ± 0.13	1.49 ± 1.44	3.83 ± 2.04	1.49 ± 0.98	0.40 ± 0.28	0.00 ± 0.00	0.60 ± 0.02	0.50 ± 0.17	0.02 ± 0.02
24	6.80 ± 2.98	1.51 ± 1.43	4.63 ± 0.92	1.68 ± 0.69	0.38 ± 0.26	0.00 ± 0.00	0.76 ± 0.23	0.60 ± 0.07	0.02 ± 0.02

Values are expressed as mean ± SEM in mM; SCFA: short-chain fatty acids; BCFA: branched-chain fatty acids; * indicates significant differences from control (Time 0 h) (*p* < 0.05); + indicates a possible trend (*p* = 0.0531).

**Table 2:** SCFA and BCFA for CH-OPT at times 0, 8, 16 and 24 h for each colonic region.

	SCFA						BCFA		
Time (h)	Acetic Acid	Propionic Acid	Butyric Acid	Valeric Acid	Caproic Acid	Heptanoic Acid	Isobutyric Acid	Isovaleric Acid	Isocaproic Acid
	Ascending colon								
0	3.96 ± 2.18	0.50 ± 0.47	2.92 ± 0.21	2.12 ± 0.05	1.80 ± 0.22	1.50 ± 0.08	4.27 ± 1.39	2.20 ± 0.09	1.56 ± 0.05
8	8.55 ± 3.71	5.05 ± 0.43	4.90 ± 0.26	4.42 ± 0.21 *	3.65 ± 0.10	2.87 ± 0.54	3.80 ± 0.22	3.20 ± 0.13	2.66 ± 0.55
16	14.12 ± 2.73	7.59 ± 0.59 *	6.97 ± 0.20 *	5.91 ± 0.37 *	4.44 ± 1.13	3.10 ± 0.36	5.19 ± 0.13	3.69 ± 0.34 *	2.80 ± 0.18
24	14.20 ± 7.02	6.53 ± 1.71 *	5.78 ± 1.21 *	5.07 ± 0.70 *	3.83 ± 0.08	3.12 ± 0.49	4.65 ± 0.61	2.81 ± 0.17^+^	2.55 ± 0.47
	Transverse colon								
0	3.27 ± 2.13	0.16 ± 0.03	1.68 ± 0.38	1.29 ± 0.21	0.89 ± 0.03	0.78 ± 0.16	2.67 ± 1.26	0.86 ± 0.04	0.64 ± 0.19
8	2.75 ± 0.41	0.82 ± 0.72	1.77 ± 0.50	1.52 ± 0.26	1.11 ± 0.47	0.76 ± 0.13	2.20 ± 0.10	0.98 ± 0.36	0.59 ± 0.10
16	2.94 ± 1.46	1.06 ± 1.00	1.37 ± 0.18	1.09 ± 0.03	0.82 ± 0.23	0.57 ± 0.08	2.20 ± 0.07	0.82 ± 0.10	0.52 ± 0.05
24	6.63 ± 2.54	1.52 ± 1.46	2.17 ± 0.18	1.84 ± 0.12	1.20 ± 0.37	0.91 ± 0.05	5.64 ± 0.87	1.09 ± 0.17	0.73 ± 0.02
	Descending colon								
0	2.43 ± 0.37	0.85 ± 0.68	2.23 ± 0.39	1.35 ± 0.17	0.83 ± 0.23	0.46 ± 0.10	1.97 ± 0.09	1.09 ± 0.07	0.40 ± 0.13
8	4.34 ± 1.07	1.21 ± 0.92	3.70 ± 0.60	2.26 ± 0.40	1.4 ± 0.0.41	0.61 ± 0.21	2.35 ± 0.11	1.76 ± 0.17	0.45 ± 0.18
16	3.84 ± 1.16	0.37 ± 0.08	3.72 ± 1.27	2.01 ± 0.05	1.34 ± 0.06	0.54 ± 0.11	3.38 ± 0.73	1.73 ± 0.15	0.52 ± 0.17
24	3.39 ± 3.39	0.43 ± 0.12	3.95 ± 1.45	2.12 ± 0.12	1.32 ± 0.01	0.54 ± 0.11	4.17 ± 0.95	1.76 ± 0.24	0.46 ± 0.13

Values are expressed as mean ± SEM in mM; SCFA: short-chain fatty acids; BCFA: branched-chain fatty acids; * indicates significant differences from control (Time 0 h) (*p* < 0.05); + indicates a possible trend (*p* = 0.0588).

## Supplementary Materials

The following are available online at https://www.mdpi.com/article/10.3390/nu13082720/s1. Figure S1: SCFA and BCFA standard curves based on peak area and concentration. Linearity was assessed using R^2^; all were above 0.99. Figure S2: Peptide profile and content. Lower molecular mass chromatograms (500-2000 m/z) of CHs before upper GI digestion. Top chromatogram CH-GL, bottom chromatogram CH-OPT. Figure S3: Peptide profile and content. Higher molecular mass chromatograms (100-5000 m/z) of CHs before upper GI digestion. Top chromatogram CH-GL, bottom chromatogram CH-OPT. Figure S4: Peptide profile and content. Lower molecular mass chromatograms (300-1500 m/z) of CHs after upper GI digestion. Top chromatogram CH-GL, bottom chromatogram CH-OPT. Figure S5: Peptide profile and content. Higher molecular mass chromatograms (1500-4000 m/z) of CHs after upper GI digestion. Top chromatogram CH-GL, bottom chromatogram CH-OPT. Table S1: List of the peptide sequences from CH-GL and CH-OPT before upper intestinal digestion. Each letter is indicative of an amino acid. Table S2: List of the peptide sequences from CH-GL and CH-OPT after upper intestinal digestion. Table S3: DPPH and FRAP for CH-GL and CH-OPT at times 0, 8, 16 and 24 h for each colonic region.
