# Supplementary material for: Gastrointestinal Digestion Model Assessment of Peptide Diversity and Microbial Fermentation Products of Collagen Hydrolysates
Source: Nutrients. 2021 Aug 7;13(8):2720. doi: 10.3390/nu13082720 (PMC8401164; doi:10.3390/nu13082720)
Supplement: Supplementary file 1 [file nutrients-13-02720-s001.zip › nutrients-1288106-supplementary.pdf]

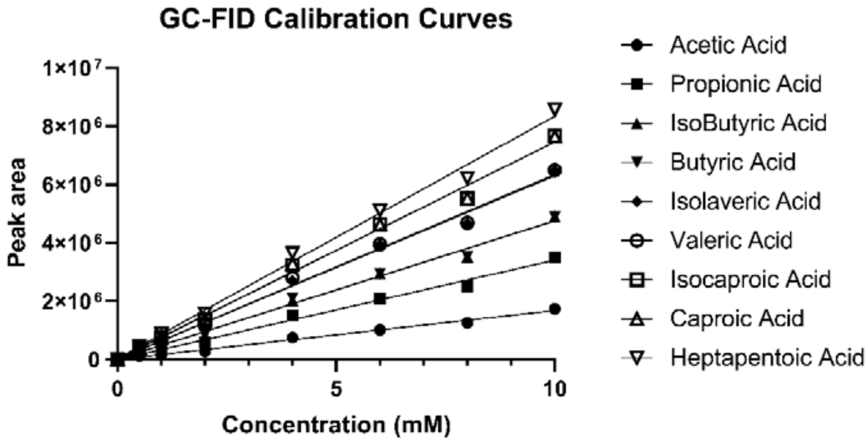


**Figure S1.** SCFA and BCFA standard curves based on peak area and concentration. Linearity was assessed using R²; all were above 0.99.

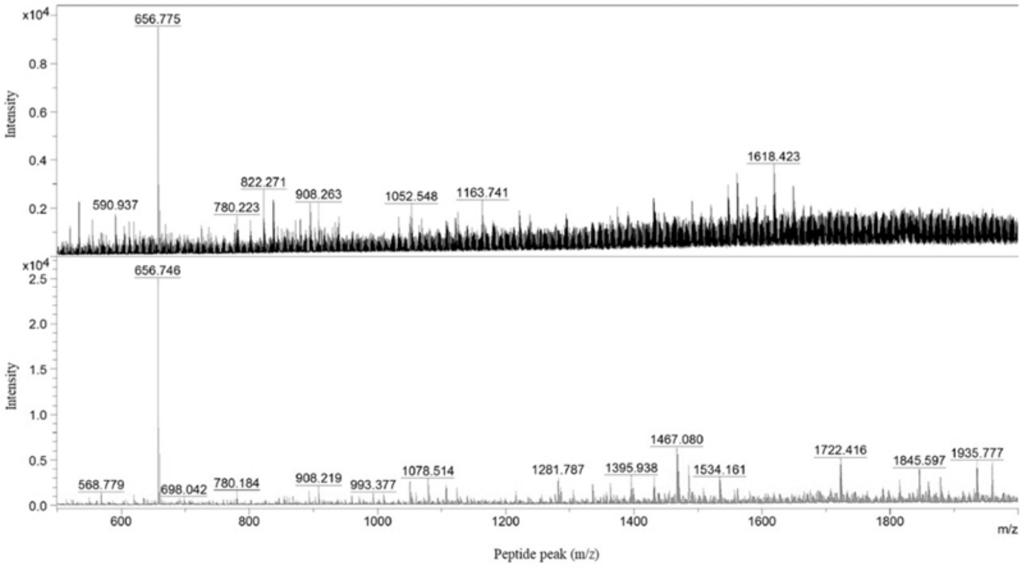


**Figure S2.** Peptide profile and content. Lower molecular mass chromatograms (500-2000 m/z) of CHs before upper GI digestion. Top chromatogram CH-GL, bottom chromatogram CH-OPT.

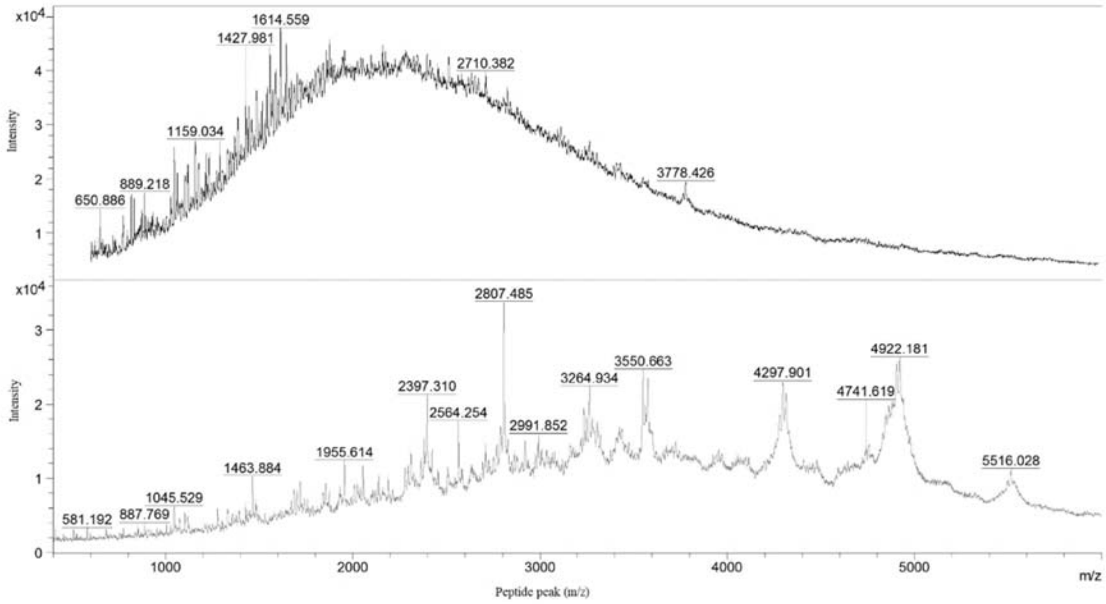


**Figure S3.** Peptide profile and content. Higher molecular mass chromatograms (100-5000 m/z) of CHs before upper GI digestion. Top chromatogram CH-GL, bottom chromatogram CH-OPT.

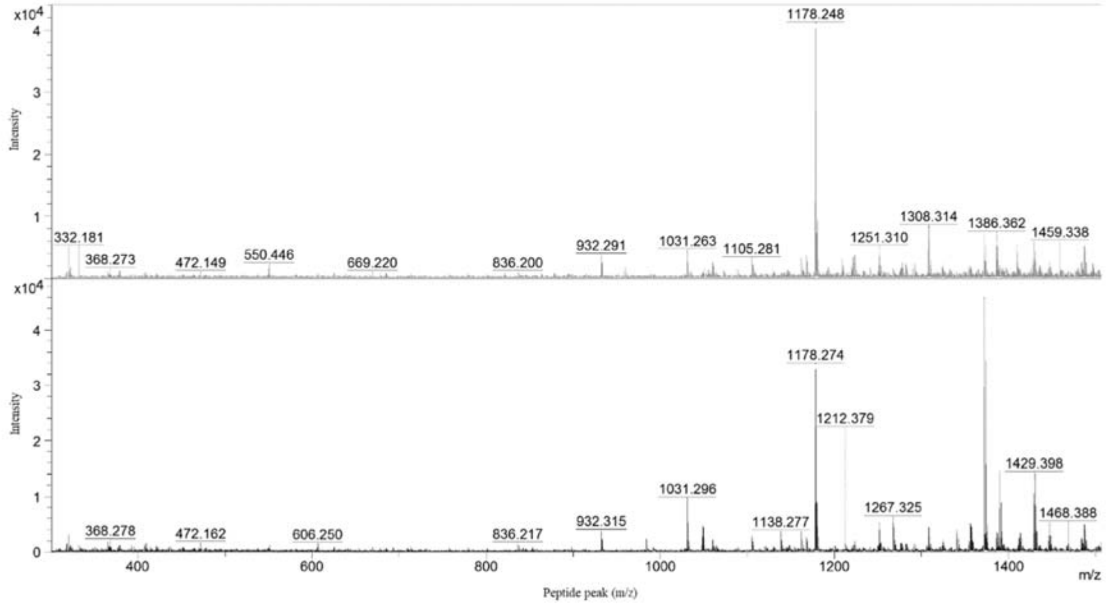


**Figure S4.** Peptide profile and content. Lower molecular mass chromatograms (300-1500 m/z) of CHs after upper GI digestion. Top chromatogram CH-GL, bottom chromatogram CH-OPT.

.

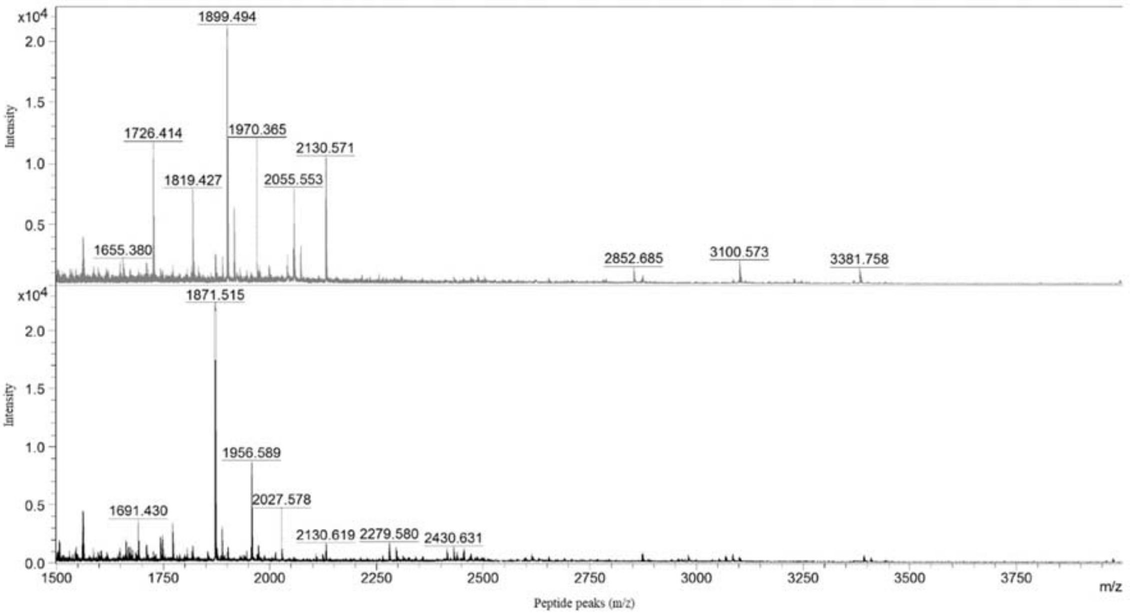


**Figure S5.** Peptide profile and content. Higher molecular mass chromatograms (1500-4000 m/z) of CHs after upper GI digestion. Top chromatogram CH-GL, bottom chromatogram CH-OPT.

**Table S1.** List of the peptide sequences from CH-GL and CH-OPT before upper intestinal digestion. Each letter is indicative of an amino acid.

| **Peptide Sequences** | | |
|---|---|---|
| CH-GL | CH-OPT | Shared sequences |
| AAGPTGPIGSR | AGPAGPAGPAGPR | AGPSGPAGPTGAR |
| AGPPGADGQPGAK | GPAGPAGPAGPR | GPAGPAGPRG |
| AGPSGPSGLPGER | GPAGPAGPAGPRG | GRPGPIGPA |
| AGPSGPSGLPGERG | GPAGPQGPR | |
| AIGPSGPAGKD | GPAGPQGPRG | |
| ARGSDGSVGPVGPA | GPMGPSGPRG | |
| AVGPAGKDGEAGAQ | GPRGPSGPQG | |
| AVGPAGPRGPAGPSGPAGKDGR | GPVGAPGRP | |
| AVGPRGPSGPQG | GPVGAPGRPG | |
| DGAPGKDGVRG | GSPGPQGPPGSIGPQ | |
| FDGDFYR | GSPGPQGPPGSIGPQG | |
| FSGLDGAKGD | PAGPQGPRG | |
| GAAGPTGPIGSR | SGPPGPPGPA | |
| GADGAPGKDGVRG | SPGPQGPPGSIGPQ | |
| GADGAPGKDGVRGL | VGSPGPAGPRG | |
| GDRGEAGPAGPAGPAGPR | VPGPMGPSGPR | |
| GDRGETGPAGPA | VPGPMGPSGPRG | |
| GDRGETGPAGPAGPIGPVGAR | | |
| GDRGETGPAGPS | | |
| GEGGPQGPRGS | | |
| GEGGPQGPRGSEGPQG | | |
| GEPGKQGPSGASGE | | |

| |
|---|
| GERGEQGPA |
| GERGFPGLPGPS |
| GGPQGPRGSEGPQG |
| GKDGEAGAQGPPGPAGPA |
| GKSGDRGETGPAGPA |
| GKSGDRGETGPAGPAGPIGPV |
| GKSGDRGETGPAGPAGPIGPVG |
| GKSGDRGETGPAGPAGPIGPVGAR |
| GPAGKSGDRGETGPAGP |
| GPAGPSGPAGKDGR |
| GPIGPVGAR |
| GPQGPPGSIGPQ |
| GPQGPPGSIGPQG |
| GPSGLPGERG |
| GPTGPIGSR |
| GPVGPSGPPGKD |
| GQGDPGRPGIPA |
| IDGRPGPIGPA |
| IGPSGPAGKD |
| IQGPPGPAGEEGKRG |
| ISVPGPMGPSGPR |
| LDGAKGDAGPAGPK |
| LGPVGNPGPAGPAGPR |
| LMGPRGPPG |
| PGDKGEAGPSGPAGPTGA |
| PGPAGPAGPR |
| QGDPGRPGIPA |
| RGPRGDQGPVGR |
| SGDRGETGPAGPA |
| SGDRGETGPAGPS |
| TAGPSGPSGLPGERG |
| TGPAGPAGPIGPVGAR |
| VGPAGPRGPA |
| VGPAGPRGPAGPS |
| VGPAGPRGPAGPSGPAGKDGR |
| VGPRGPSGPQG |
| VMGPAGSRG |
| VQGPPGPAGPR |
| VQGPPGRPGPQ |
| VVGLPGQR |

**Table S2.** List of the peptide sequences from CH-GL and CH-OPT after upper intestinal digestion.

| Peptide Sequences | | |
|---|---|---|
| CH-GL | CH-OPT | Shared Sequences |
| AAGPPGPTGPAGPPGFPGAVGAK | AAGEPGKAGER | AAGAPGPQGPVGPVGK |
| AAGPPGPTGPAGPPGFPGAVGAKGEGGPQGPR | AAGEPGKNGAK | AAGLPGVAGAPGLPGPR |
| AFLSIHS | AAGLPGPKGDRGDAGPK | AGAPGAPGSQGAPGLQGMPGER |
| AGPPGLLGPPGPR | AAGPSGPNGLPAGSR | AGAPGPQGPVGPVGK |
| AGPPGPTGPAGPPGFPG | AAGPTGPIGSR | AGEAGKPGER |
| ANGIPGPIGPPGPR | AAGPTGPIGSRG | AGLPGVAGAPGLPGPR |
| APGAPGPVGPA | AAGQPGAKGER | AGPPGFPGAPGPK |
| AVGPAGAVGPR | ADGPAGAPGTPGPQG | AGPPGPTGPAGPPGFPGAVGAK |
| DARPNSWPWQ | AGAPGIPGGK | AGPPGPTGPAGPPGFPGAVGAKGEGGPQGPR |
| DGANGIPGPIGPPGPR | AGAPGIPGGKGDSGAPGER | AGPSGPSGLPGER |
| DGATGAAGPPGPTGPAGPPGFPGAVGAK | AGAPGLPGPR | AGPSGPSGLPGERG |
| DGDPGLRGPPGLPGPL | AGAPGPPGPPGSDGSPGGK | DGEAGAQGPPGPAGPAGER |
| DGIPGQPGLPGP | AGEDGHPGKPGRPGE | DGLNGLPGPIGPPGPR |
| DGLPGLPGLPGPP | AGEDGHPGKPGRPGERG | DGLNGLPGPIGPPGPRG |
| DGRPGPIGP | AGEPGKAGER | DGPRGPTGPIGPPGPA |
| DGRPGPIGPA | AGEPGRDGNPGSDGLPGR | DKGEPGDKGPR |
| DGSPGAKGDRGETGPAGPPGAPGAPGAPGPVGPAGK | AGFPGSPGAK | DTEYFGTIGIGTPAQDF |
| DLGPRGPPGIPGREGPK | AGPAGAAGQPGAKGER | EGPVGLPGIDGRPGPIGPA |
| DLSFLPQPPQ | AGPAGPAGPAGPR | ERGPPGNPGLP |
| DLSFLPQPPQE | AGPAGPKGEPGSPGENGAPGQMGPR | EVGPRGLPGEPGP |
| DNIWDQGLVSQDL | AGPAGPPGPPGAIGPSGPAGK | FGLPGPAGAR |
| DQGLVSQDLFS | AGPIGSAGPPGFPG | FQGPPGEPGEPGASGPMGPR |
| DRGETGPAGPAGPIGPVG | AGPPGADGQPGA | FRNVVDGQPF |
| DRGETGPAGPAGPIGPVGA | AGPPGADGQPGAK | GAAGEPGKAGER |
| DTEYFGTIGIGTPAQD | AGPPGESGR | GAAGLPGVAGAPGLPGPR |
| DTGSSNLWVPS | AGPPGPAGPAGPPGP | GANGAPGIAGAPGFPGAR |
| DTVQVGGISDTNQIFG | AGPPGPPGPAGK | GAPGDRGEPGPPGPAGF |
| EFGFDGDFYR | AGPPGPSGPPGEK | GAPGPQGPVGPVGK |
| EFGFDGDFYRA | AGPTGPIGSR | GAPGTAGPSGPSGLPGER |
| EGIGKPGAP | AGPTGPIGSRG | GARGEPGPAGLPGPPGE |
| EGPVGLPGIDGR | AGQPGAKGER | GDGGPPGATGFPGAAG |
| EGPVGLPGIDGRPGPI | AGRPGEAGLPGAK | GDRGETGPAGPAGPIGPV |

| | | |
|---|---|---|
| EGPVGLPGIDGRPGPIG | AGSPGKDGLNGLPGPIGPPGPR | GDRGETGPAGPAGPIGPVG |
| EGPVGLPGIDGRPGPIGPAG | AIGFPGPK | GDRGETGPAGPAGPIGPVGA |
| EGPVGLPGIDGRPGPIGPAGA | AIGPSGPAGK | GDRGETGPAGPPGAPGAPGAPGPVGPAG |
| EGPVGLPGIDGRPGPIGPAGAR | AKGDPGPR | GEPGNIGFPGPK |
| EGSPGPLGPIGPPGVR | AKGEPGDAGAK | GEPGPAGAVGPAGAVGPR |
| EPGPLGIAGPPGAR | ANGLPGEKGPPGDR | GEPGPAGLPGPP |
| ERGPPGPMGPPGLAGPPGESGR | APGPKGAR | GEPGPAGLPGPPG |
| ETGPAGPAGPIGPV | AQGPPGPAGPAGER | GEPGPAGLPGPPGE |
| ETGPAGPAGPIGPVG | ARGEPGPAGLPGPPGER | GEPGPAGLPGPPGER |
| ETGPAGPAGPIGPVGA | ARGPAGPQGPR | GEPGPLGIAGPPGAR |
| FDGDFYR | ARGPPGPPGK | GEPGPTGIQGPPGPAGEEGK |
| FDNIWDQGLVS | ARGPPGPPGTNGVPGQR | GEQGPAGSPGF |
| FDNIWDQGLVSQ | ARGPSGPQGPSGPPGPK | GERGPPGESGAAGPTGPIGS |
| FGFDGDFYR | ARGPSGPQGPSGPPGPKGNSGEPGAPGSK | GETGPAGPAGPIGPVG |
| FGFDGDFYRA | ARGSDGSVGPVGPA | GFPGADGVAGPK |
| FGTIGIGTPA | ARGSDGSVGPVGPAGP | GFPGADGVAGPKGPAGE |
| FGTIGIGTPAQD | ASGHPGPIGPPGPR | GFPGADGVAGPKGPAGER |
| FLGLPGSR | ASGPAGPRGPPGSAGSPGK | GFPGLPGPSGEPGK |
| FPGERGLPGA | ASGPAGPRGPPGSAGSPGKDG | GFPGLPGPSGEPGKQ |
| FPGLPGPSGEPGK | ASGPMGPR | GFPGLPGPSGEPGKQGAPGA |
| FPGPKGANGEPGK | AVAGPPGPSGPPGEK | GFPGLPGPSGEPGKQGPSGA |
| FPGYPGPK | AVGPAGKDGEAG | GFPGLPGPSGEPGKQGPSGASGER |
| GAAGPPGPTGPAGPPGFPGAVGAK | AVGPAGKDGEAGAQ | GFQGPPGEPGEPGASGPMGPR |
| GAPGAIGAPGPAGANGDRGEAGPAGPAGPAGPR | AVGQPGPPGPSGEEGK | GFSGLDGAK |
| GAPGFLGLPGSR | AVGSPGPAGPR | GFSGLDGAKGDAGPAGPK |
| GAPGFPGPK | DGAKGDAGPAGPK | GFSGLQGPPGPPGSPGEQGPSGASGPAGPR |
| GAPGIAGAPGFPGAR | DGAKGDAGPAGPKGEPGSPGENGAPGQMGPR | GHGAGGASIL |
| GAVGPAGAVGPR | DGASGHPGPIGPPGPR | GHNGLDGL |
| GDAGPPGPAGPAGPPGPIG | DGEAGAQGPPGPAGPA | GIDGRPGPIGPA |
| GDGGPPGATGFPGAAGR | DGEAGAQGPPGPAGPAGE | GIPGPFGPR |
| GDLGPRGPPGIPGREGPK | DGEAGAQGPPGPAGPAGERGEQGPAGSPGF | GIRGPPGLP |
| GDPGMPGLPGLKGDEGIQGLP | DGNPGSDGLPGR | GISVPGPMGPSGPR |

| | | |
|---|---|---|
| GDRGETGPAGPPGAPGAPGAPGPVGPAGK | DGPRGPTGP | GLNGLPGPIGPPGPR |
| GDTGAKGEPGPTGIQGPPGPAGEEGK | DGRPGPPGPPGAR | GLPGAKGAAGLPGVAGAPGLPGPR |
| GDVGPLGPLGKEGPPGPR | DGSPGAKGDR | GLPGLKGHNGLQGLPGL |
| GEIGPAGPPGPPGLR | DGSPGAKGDRGETGPAGPPGAPGAPGAPGPVGPA | GLPGLKGHNGLQGLPGLA |
| GENGVPGEDGAPGPMGPR | EAGPAGPAGPAGPR | GLPGPIGPPGPR |
| GEPGPPGLDQP | EGAPGAEGSPG | GLPGPPGAPGPQGF |
| GEPGPTGIQGPPGPAGEEGKR | EGAPGAEGSPGR | GLPGPPGAPGPQGFQGPPGEPGEPGASGPMGPR |
| GETGPAGPAGPIGPV | EGAPGAEGSPGRDGSPGA | GLPGVAGSVGEPGPLGIAGPPGA |
| GETGPAGPAGPIGPVGA | EGAPGAEGSPGRDGSPGAK | GLPGVAGSVGEPGPLGIAGPPGAR |
| GETGPAGPAGPIGPVGAR | EGLRGPR | GLTGPIGPPGPAGAPGDK |
| GETGPAGPPGAPGAPGAPGPVGPAGK | EGPQGPPGPVGSPGER | GNDGATGAAGPPGPTGPAGPPGFPGAVGAK |
| GEVGFQGKTGPPGPPG | EGSKGPRG | GPAGLPGPPGER |
| GEVGLPGLSGPVGPPGNPGANGLPGAKGAAGLPGVAGAPGLPGPR | EPGSSGVDGAPGKDGPRGPTGPIGPPGPAGQPGDK | GPIGPPGPR |
| GEVGPAGPNGF | FAGPAGAAGQPGAKGER | GPPGEPGEPGASGPMGPR |
| GFDGDFYRA | FAGPPGADGQPGAK | GPPGESGAAGPTGPIGS |
| GFDGLDFDWEYPGSR | FDNIWDQGLVSQDL | GPPGESGAAGPTGPIGSR |
| GFLLPASQIIPTAQ | FLPQPPQEK | GPPGPMGPPG |
| GFMPSDR | FSGLDGAK | GPPGPMGPPGL |
| GFPGLPGPS | FSGLDGAKG | GPPGPMGPPGLAGPPGESG |
| GFPGPPGPIGL | FSGLDGAKGD | GPPGPMGPPGLAGPPGESGR |
| GFPGPPGPK | GAAGEPGKAGE | GPPGPPGPPGPPGPPSG |
| GFPGSPGNIGPAGK | GAAGEPGKNGAK | GPPGPPGPPGPPGPPSGGY |
| GFPGTPGLPGFK | GAAGIPGGKGEK | GPPGPPGPPGPPGPPSGGYD |
| GFPGTPGLPGFKG | GAAGIPGGKGEKGETGL | GPPGPSGLPGLP |
| GFPGTPGLPGFKGI | GAAGLPGPK | GPPGSAGSPGKDGLNGLPGPIGPPGPR |
| GFPGTPGLPGFKGIR | GAAGLPGPKGD | GPPGSAGSPGKDGLNGLPGPIGPPGPRG |
| GFPGYPGPK | GAAGLPGPKGDR | GPSGPSGLPGER |
| GGGYEFGFDGDF | GAAGLPGPKGDRGD | GPSGPSGLPGERG |
| GGGYEFGFDGDFYR | GAAGLPGPKGDRGDAGPK | GPTGPIGPPGPA |
| GGISDTNQIF | GAAGPTGPIGSR | GRTGDAGPAGPPGPPGPPGPPGPPS |
| GGISDTNQIFG | GAAGQPGAKGER | GRTGDAGPAGPPGPPGPPGPPGPPSG |
| GGPGPMGLMGPR | GADGAPGKDG | GSAGPPGATGFPGAAG |

| | | |
|---|---|---|
| GGYDLSFLPQPPQE | GADGAPGKDGV | GSAGPPGATGFPGAAGR |
| GGYEFGFDGDF | GADGAPGKDGVR | GSPGERGEVGPAGPNGF |
| GHPGPPGPVGPA | GADGAPGKDGVRG | GSVGEPGPLGIAGPPGAR |
| GIDGRPGPIGP | GAKGDAGAPGAPGSQGAPGLQGMPGER | GVPGDLGAPGPSGAR |
| GIDGRPGPIGPAGA | GAKGEPGDAGAK | GVPGPPGAVGPAGK |
| GIPGEFGLPGPAGA | GANGAPGNDGAK | GVPGPPGAVGPAGKDGEAGAQGPPGPAGPAGE |
| GIPGEFGLPGPAGAR | GANGAPGNDGAKGDAGAPGAPGSQGAPGLQGMPGER | GVPGPPGAVGPAGKDGEAGAQGPPGPAGPAGER |
| GIPGEFGLPGPAGARGER | GANGDRGEAGPAGPAGPAGPR | GVVGLPGQR |
| GIPGLPGPR | GAPGADGPAGAPG | HMWPGDIK |
| GIPGLPGPRG | GAPGADGPAGAPGTPGPQ | IDGRPGPIGP |
| GIPGPVGAAGATGAR | GAPGADGPAGAPGTPGPQG | IDGRPGPIGPA |
| GISVPGPMGPSGPRG | GAPGADGPAGAPGTPGPQGIAGQ | IGFPGPKGPSGDPGK |
| GIVGLPGQR | GAPGAIGAPGPA | IGPAGPPGPPGLR |
| GLLGLPGQPG | GAPGAPGAPGPVGPA | ILGDVFIR |
| GLLGPKGPPGIPGPPGV | GAPGAPGPVGPA | ISVPGPMGPSGPR |
| GLLGPKGPPGPPGPPGVTGM | GAPGAVGPAGPR | LNGLPGPIGPPGPR |
| GLPGADGRAGVMGPAGSR | GAPGDKGEAGPSGPAGPT | LPGPIGPPGPR |
| GLPGEMGRP | GAPGDRGEPGPPGPA | LQGPPGPPGSPGEQGPSGASGPAGPR |
| GLPGLKGHNGLQGLPGLAGHHGDQGAPGAVGPAGPR | GAPGDRGEPGPPGPAG | LSFLPQPPQE |
| GLPGLPGEKGHT | GAPGEKGEGGPPGAAGPA | NGDDGEAGKPGRPGER |
| GLPGLPGPK | GAPGFPGPR | NGLPGPIGPPGPR |
| GLPGPAGPPGE | GAPGIPGGK | PGADGVAGPK |
| GLPGPIGPKG | GAPGNDGAK | PGFGLPGK |
| GLPGPIGPPGPRG | GAPGPAGPK | PGPMGPSGPR |
| GLPGPLGPPGP | GAPGPAGPKGSPGEAGRPGEAGLPGA | QGLPGPAGPPGEAGKPGEQGVPGDLGAPGPSGAR |
| GLPGPPGAP | GAPGPAGPKGSPGEAGRPGEAGLPGAK | QGPPGEPGEPGASGPMGPR |
| GLPGPPGAPGP | GAPGPAGPKGSPGEAGRPGEAGLPGAKG | RGEPGNIGFPGPK |
| GLPGTAGLPGMK | GAPGPAGSRGPPGPQGPR | RGETGPAGPAGPIGPVG |
| GLPGVAGAPGLPGPR | GAPGPMGPR | RGETGPAGPAGPIGPVGA |
| GLPGVAGSVGEPGPL | GAPGPQGPPGAPGP | RGPPGPMGPPGLAGPPGESGR |
| GLPGVAGSVGEPGPLGI | GAPGPQGPPGAPGPL | SGATPVFDNIWDQGLVSQDL |
| GLQGLPGLAGHHGDQGAPGAVGPAGPR | GAPGPQGPPGAPGPLG | SGDRGETGPAGPAGPIGPV |
| GLSGLPGPPGPPGPR | GAPGSKGDTGAK | SGDRGETGPAGPAGPIGPVG |
| GLTGPIGPPGPAGAPGDKGEAGPSGPAGPT | GAPPGEGPGEQK | SGDRGETGPAGPAGPIGPVGA |

| | | |
|---|---|---|
| GLTGPIGPPGPAGAPGDKGEAGPSGPAGPTGA | GARGEPGPAGLPGPPGER | SGLQGPPGPPGSPGEQGPSGASGPAGPR |
| GLTGPIGPPGPAGAPGDKGEAGPSGPAGPTGAR | GARGEPGPAGLPGPPGERGGPGS | SVPGPMGPSGPR |
| GNIGFPGPK | GASGHPGPIGPPGPR | TGDAGPAGPPGPPGPPGPPGPPS |
| GNPGQPGQPGLP | GAVGQPGPPGPSGEEGK | TGISVPGPMGPSGPR |
| GPAGLPGVPGR | GDAGAPGAPGSQGAPGLQGMPGER | TGPPGPSGISGPPGPPGPAGK |
| GPAGPAGPIGP | GDAGPAGPK | VALRGPPGP |
| GPAGPAGPIGPV | GDAGPAGPKGEPGSPGENGAPGQMGPR | VGGISDTNQIF |
| GPAGPAGPIGPVG | GDAGPPGPAGPA | VPGPMGPSGPR |
| GPAGPAGPIGPVGA | GDAGPPGPAGPAGPPGP | |
| GPAGPIGPV | GDAGPPGPAGPAGPPGPI | |
| GPAGPIGPVG | GDAGPPGPAGPAGPPGPIGN | |
| GPAGPIGPVGA | GDIGSPGRDGA | |
| GPAGPPGLLGP | GDKGEPGDKGPR | |
| GPAGPPGLLGPPGPR | GDKGEPGSSGVDGAPGK | |
| GPAGPPGLLGPPGPRG | GDKGEPGSSGVDGAPGKDGPR | |
| GPAGPPGPPGLR | GDKGEQGPPGPTGPQGPIG | |
| GPAGPPGPPGLRG | GDKGETGEQGDR | |
| GPGPMGLMGPR | GDKGETGEQGDRG | |
| GPIGFPGPK | GDQGAPGAVGPAGPR | |
| GPIGPPGIP | GDRGDAGPK | |
| GPIGPPGIPGPK | GDRGDAGPKGADGAPGKDGV | |
| GPIGPPGPRG | GDRGEAGPA | |
| GPIGPPGRP | GDRGEAGPAGPA | |
| GPMGLMGPR | GDRGEAGPAGPAGPAGPR | |
| GPPGAPGLPG | GDRGENGSPGAPGAPGHPGPPGPVGPA | |
| GPPGAPGLPGQ | GDRGETGPAGPA | |
| GPPGAPGPLGI | GDRGETGPAGPAGPIGP | |
| GPPGASGAPGPQGF | GDRGETGPAGPPGAP | |
| GPPGEPGEPGA | GDRGETGPAGPPGAPGAPGAPGPVGPA | |
| GPPGFGLPGK | GDRGETGPAGPSGAPGPAGSR | |
| GPPGFPGPK | GDSGAPGERGPPGAGGPPGPR | |
| GPPGFQGLP | GDSGAPGERGPPGAGGPPGPRG | |
| GPPGHPGLP | GDSGPPGER | |
| GPPGLLGPPGPR | GDSGPPGERGAPGPQG | |
| GPPGLPGLPGFK | GDSGRPGEPGLMGPR | |
| GPPGLPPPRVVGASP | GEAGAPGIPGGK | |
| GPPGPPGFPGKP | GEAGAPGIPGGKGDSGAPGER | |
| GPPGPPGLPGPK | GEAGAPGIPGGKGDSGAPGERGPPG | |

| | | |
|---|---|---|
| GPPGPPGPPGPK | GEAGAPGIPGGKGDSGAPGERGPPGA GGPPGPR | |
| GPPGPPGPPGPPGLR | GEAGAQGPPGPAGPAGER | |
| GPPGPPGPPGPPGPPS | GEAGKPGER | |
| GPPGPPGPPGPPGPPSGGYDL | GEAGPAGPAGPAGPR | |
| GPPGPTGPAGPPGFPG | GEAGSPGIAGPK | |
| GPPGQPGLP | GEDGKDGSPGEPGANGLPG | |
| GPSGPQGPSGPPGP | GELGPVGNPGPAGPAGPR | |
| GPTGLPGPAGPPGL | GENGPVGPTGPVGAAGPSGPNGPPG PAGSR | |
| GPTGPIGPPGP | GEPGAKGER | |
| GPTGPIGPPGPAG | GEPGAVGQPGPPGPSGEEGK | |
| GPVGPAGPIG | GEPGDAGAK | |
| GPVGPTGPVGAAGPSGPNGPPGPAG SR | GEPGPAGLPGPPGERGGPGS | |
| GRPGAPGPAGAR | GEPGPPGQP | |
| GRPGLPGPI | GEPGPQGH | |
| GSDGSVGPVGPAGPI | GEPGPRGPPGA | |
| GSDGSVGPVGPAGPIG | GEPGRDGNPGSDGLPGR | |
| GSDGSVGPVGPAGPIGSAGPPG | GEPGSPGENGAPGQMGPR | |
| GSDGSVGPVGPAGPIGSAGPPGF | GEQGPAGPPGFQ | |
| GSDGSVGPVGPAGPIGSAGPPGFPGA PGPK | GEQGPPGPAGFPGAPGQNGEPGAK | |
| GSDGSVGPVGPAGPIGSAGPPGFPGA PGPKGEL | GEQGPSGASGPAGPR | |
| GSTGEIGPAGPPGPPGL | GERGEAGSPGIAGPK | |
| GSTGEIGPAGPPGPPGLR | GERGPPGESG | |
| GSVGPVGPAGPIGSAGPPGFPGAPGP K | GERGPPGESGAAGPT | |
| GTAGPSGPSGLPGER | GERGPPGESGAAGPTGPIG | |
| GTGSMTGILGYDTVQVGGI | GERGPPGESGAAGPTGPIGSR | |
| GTGSMTGILGYDTVQVGGISDTNQ | GERGPPGNPGLP | |
| GTGSMTGILGYDTVQVGGISDTNQIF G | GERGSPGGP | |
| GVDGLPGQPGPR | GESGAPGVPGIAGPR | |
| GVMGFPGPK | GESGNKGEPGAVGQPGPPGPSGEEGK | |
| GVMQGPMGPMGPR | GETGAPGLKGENGVPGENGAPGPMG PR | |
| GVPGPPGAVGPAGKDGEA | GETGPAGPPG | |
| GYDLSFLPQPPQ | GETGPAGPPGAPGAPGAPGPVGPA | |
| GYDTVQVGGI | GETGPAGPSGAPGPAGSR | |
| GYPGNAGPVGAAGAPGPQGPVGPV GK | GETGPAGRPGEVGPPGPPGPA | |
| IAGAPGFPGAR | GETGPQGPPGPTGPSGDKGD | |
| IDGRPGPIGPAG | GEVGAPGDPG | |

| | | |
|---|---|---|
| IGQPGAVGPAGI | GEVGPAGPNGFAGPAGAAGQPGAKGE | |
| IGQPGAVGPAGIR | GEVGPAGPNGFAGPAGAAGQPGAKGER | |
| IGQPGAVGPAGIRG | GEVGPRGLPGEPGP | |
| ILGDVFIRQ | GFMPSDRA | |
| ILGDVFIRQY | GFPGAPGQNGEPGAK | |
| ILGPPGPR | GFPGLPGPSGEPGKQGAPG | |
| IPGLPGVPGPRGP | GFPGLPGPSGEPGKQGAPGAS | |
| ISGPSGPIGPPGIPGPK | GFPGLPGPSGEPGKQGPS | |
| ISVPGPMGPSGPRG | GFPGLPGPSGEPGKQGPSG | |
| KGEPGVVGAPGTAGPSGPSGLPGER | GFPGLPGPSGEPGKQGPSGAS | |
| KNWGEGW | GFPGLPGPSGEPGKQGPSGASGE | |
| LDTEYFGTIGIGTPAQDF | GFPGNPGAPGSPGPA | |
| LGAPGFLGLPGSR | GFPGNPGAPGSPGPAGH | |
| LGFPGER | GFPGNPGAPGSPGPAGHQGAVGSPGPAGPR | |
| LGPLGAPGEDGRPGPPGSIGI | GFPGPKGAAGEPGKAGER | |
| LGSLGSPGLPGLPGPPG | GFPGPPGMKGPA | |
| LGVPGLPGYPGR | GFPGSPGAK | |
| LPASQIIPTAQ | GFSGLDGAKGD | |
| LPGDRGPPGPPGI | GFSGLDGAKGDAGPAGPKGEPGSPGENGAPGQMGPR | |
| LPGLPGPK | GGAGPPGPEGGK | |
| LTGPIGPPGPAGAPGDKGEAGPSGPAGPTGAR | GGPGERGEQGPPGPA | |
| MGFPGPK | GGPGERGEQGPPGPAGFPGAPGQNGEPGAK | |
| MGIKGHRGFPGNPGAPGSPGPAGHQGAVGSPGPAGPR | GGPGERGEQGPPGPAGFPGAPGQNGEPGAKGE | |
| MGPPGEPGLPG | GGPGGPGPKGDKGEPGSSGVDGAPGK | |
| MMGPPGPPGPLGIPGR | GGPGGPGPQGPAG | |
| MQGPMGPMGPR | GGPGGPGPQGPAGK | |
| MWPGDIK | GGPGPAGPR | |
| PAGPAGPIGPV | GGPGSRGFPGADGVAGPK | |
| PAGPAGPIGPVG | GGQGPPGSPGPK | |
| PAGPAGPIGPVGA | GHNGLDGLKGQPGAPGVKGEPGAPGENGTPGQTGA | |
| PAGPPGLLGPPGPR | GHNGLQGLPGLAGH | |
| PGADGVAGPKGPAGER | GHPGPIGPPGPR | |
| PGAPGFPGPK | GIAGPPGAR | |
| PGEPGEPGVSGPMGPR | GIPGPVGEQGLPGAP | |
| PGEPGPQGPIGVP | GIQGPPGPAGEEGK | |
| PGFPGLPGK | GISGPPGPPGPAGK | |
| PGGPPGLPGPAGPKGAK | GIVGLPGQRGE | |

| | | |
|---|---|---|
| PGLAGSPGLPGIHGLQ | GKDGEAGAQ | |
| PGLHGLPGEKGDPGPPGFDVSGPPGERGSPGIPGAPGPMGPPGSPGLPGK | GKEGSKGPR | |
| PGLPGGQGPPGSPGPKGSPGFPGIPGPPGQPGPR | GKSGDRGETGPAGPA | |
| PGPMGLMGPR | GKSGDRGETGPAGPAGP | |
| PGPPGPPGPPGLR | GKSGDRGETGPAGPAGPIGP | |
| PGPQGPPGL | GKSGDRGETGPAGPAGPIGPV | |
| PGPQGPPGLA | GKSGDRGETGPAGPAGPIGPVG | |
| PGPSGISGPPGPPGPAGKEGLR | GKSGDRGETGPAGPAGPIGPVGA | |
| PGPVGAAGATGAR | GLAGPAGPQ | |
| PGTPGLPGFKGIR | GLAGPPGESGR | |
| PGVVGAPGTAGPSGPSGLPGER | GLMPGSVGPVGPR | |
| PIGFPGPK | GLPGAAGER | |
| PMGLMGPR | GLPGADGR | |
| PPGFGLPGK | GLPGADGRA | |
| PPGLLGPPGPR | GLPGADGRAG | |
| PQGLPGLPGEP | GLPGADGRAGVM | |
| PTGFPGPK | GLPGAPGPR | |
| PVFDNIWDQGLVS | GLPGEKGPPGDR | |
| QGPSGASGERGPPGPMGPPGL | GLPGEKGPPGDRGGPGPAGPR | |
| QGPSGASGERGPPGPMGPPGLAGPPGESGR | GLPGEPGPR | |
| QVGGISDTNQIF | GLPGGGPPGLGLPGPKG | |
| QVIGGEGPGLPGQKGDPGPPGPPGSR | GLPGLKGH | |
| RGEPGPAGAVGPAGAVGPR | GLPGLKGHNGL | |
| RGETGPAGPAGPIGPV | GLPGLKGHNGLQGLPG | |
| RGETGPAGPAGPIGPVGAR | GLPGLKGHNGLQGLPGLAG | |
| RGETGPAGPPGAPGAPGAPGPVGPAGK | GLPGLKGHNGLQGLPGLAGH | |
| RGPVGPPGAP | GLPGPKGDR | |
| RGSTGEIGPAGPPGPPGL | GLPGPKGDRGDAGPK | |
| RGSTGEIGPAGPPGPPGLR | GLPGPPGAPGPQ | |
| RNVVDGQPF | GLPGPPGAPGPQG | |
| RPGPPGPPGPK | GLPGPPGER | |
| SAGPPGFPGAPGPK | GLPGPRGPQ | |
| SGATPVFDNIWDQGLV | GLPGPSGEPGK | |
| SGDRGETGPAGPAGPIG | GLPGQPGIPGE | |
| SGDRGETGPAGPAGPIGPVGAR | GLPGTSGPPGENGKPGEPGPK | |
| SGETGASGPPGF | GLQGPPGPPGSPGEQGPSGASGPAGPR | |
| STGISVPGPMGPSGPR | GLTGPIGPPGPA | |
| SVGPVGPAGPIGSAGPPGFPGAPGPK | GLTGSPGSPGPDG | |
| TAGPSGPSGLPGER | GLTGSPGSPGPDGK | |

| | | |
|---|---|---|
| TAILPGLGR | GLVGEPGPAGSK | |
| TGPAGPAGPIGPV | GNAGPPGPPGPAGK | |
| TGPAGPAGPIGPVG | GNDGATGAAGPPGPTGPAGPPGF | |
| TGPAGPAGPIGPVGAR | GNPGPAGPAGPR | |
| TGPIGPPGPAGAPGDKGEAGPSGPA GPTGAR | GNPGSDGLPGR | |
| TGPPGPSGISGPPGPPGPAGKEGL | GNSGEPGAPGSK | |
| TVQVGGISDTNQIF | GNSGEPGAPGSKGDTGAK | |
| VGEPGPLGIAGPPGAR | GPAGANGLPGEK | |
| VGGISDTNQI | GPAGAPGTPGPQ | |
| VGGISDTNQIFG | GPAGAPGTPGPQG | |
| VGLMPGSVGPVGPR | GPAGPAGPAGPR | |
| VMGFPGPK | GPAGPAGPR | |
| VPGEDGAPGPMGPR | GPAGPPGLPGSVGAPGPR | |
| VPGLPGYPGR | GPAGPPGPAGE | |
| VPGPMGPSGPRG | GPAGPPGPIGN | |
| VPVVTGIR | GPAGPPGPR | |
| VVGGEDARPNSWPWQ | GPAGPQGPR | |
| WGFMPSDRA | GPAGPQGPRGD | |
| YDLSFLPQPPQE | GPAGPQGPRGDKGETGEQ | |
| YEFGFDGDFYRA | GPAGPQGPRGDKGETGEQGD | |
| | GPAGPQGPRGDKGETGEQGDR | |
| | GPAGPSGPAGK | |
| | GPAGPSGPAGKDG | |
| | GPAGPSGPAGKDGR | |
| | GPAGSRGATGPAGV | |
| | GPAPGAADGGPQ | |
| | GPEGLPGPQGPK | |
| | GPIGPPGRA | |
| | GPKGENGPVGPTGPV | |
| | GPKGENGPVGPTGPVG | |
| | GPKGENGPVGPTGPVGAAGPSGPNG PPGPAGS | |
| | GPKGENGPVGPTGPVGAAGPSGPNG PPGPAGSRGDGGPPGATGFPGAAG | |
| | GPKGPPGPPGL | |
| | GPKGPPGPPGP | |
| | GPNGDSGRPGEPGLM | |
| | GPPGAGGPPGPR | |
| | GPPGAGGPPGPRG | |
| | GPPGDPGLPG | |
| | GPPGDRGGPGPAGPRG | |
| | GPPGEPGLPG | |
| | GPPGFPGAPGPK | |

| | | |
|---|---|---|
| | GPPGKDGASGHPGPIGPPGPR | |
| | GPPGLAGPPGE | |
| | GPPGLAGPPGESGR | |
| | GPPGPAGEEGK | |
| | GPPGPAGPAGER | |
| | GPPGPAGPAGERGEQGPA | |
| | GPPGPAGPR | |
| | GPPGPEGPR | |
| | GPPGPMGPPGLA | |
| | GPPGPMGPPGLAGPPGESGRE | |
| | GPPGPPGAIGPSGPAGK | |
| | GPPGPPGIR | |
| | GPPGPPGKR | |
| | GPPGPPGLPG | |
| | GPPGPPGLR | |
| | GPPGPPGPAALPGSK | |
| | GPPGPPGPAGK | |
| | GPPGPQGLPGLAG | |
| | GPPGPQGPR | |
| | GPPGPSGEAGPPGPPGK | |
| | GPPGPVGPPG | |
| | GPPGPVGPPGLKGDS | |
| | GPPGQPGLPG | |
| | GPPGSAGSPGK | |
| | GPPGSAGSPGKDG | |
| | GPPGSAGSPGKDGL | |
| | GPRGPPGPAGA | |
| | GPSGDPGKAGEK | |
| | GPSGDPGKAGEKGH | |
| | GPSGEPGTAGPPGTPGPQG | |
| | GPSGLPGER | |
| | GPSGLPGERG | |
| | GPSGPNGPPGPAGSR | |
| | GPSGPPGPDGNKGEPG | |
| | GPSGPPGPDGNKGEPGV | |
| | GPSGPPGPDGNKGEPGVVGAPG | |
| | GPSGPPGPDGNKGEPGVVGAPGTAG PSGPSGLPGERG | |
| | GPSGPQGIR | |
| | GPSGPQGPSGPPGPK | |
| | GPSGPQGPSGPPGPKGN | |
| | GPSGPQGPSGPPGPKGNSGEPGAPGS K | |

| | | |
|---|---|---|
| | GPTGPIGPPGPAGQPGD | |
| | GPTGPIGPPGPAGQPGDKG | |
| | GPVGNPGPAGPAGPR | |
| | GPVGPSGPPGK | |
| | GPVGPSGPPGKD | |
| | GPVGPSGPPGKDGAS | |
| | GPVGPSGPPGKDGASGHPGP | |
| | GPVGPSGPPGKDGASGHPGPIGPPGP R | |
| | GPVGPTGPVG | |
| | GPVGPTGPVGA | |
| | GPVGPTGPVGAA | |
| | GQAGVMGFPGPK | |
| | GQPGAKGER | |
| | GQPGPPGPPGTAGFPGSPGAK | |
| | GQPGVMGFPGPK | |
| | GRPGAPGPA | |
| | GRPGAPGPAGA | |
| | GRPGEVGPPGPPGPAGEK | |
| | GRPGLPGAAGA | |
| | GRPGPIGPAGA | |
| | GRPGPPGPPG | |
| | GRTGDAGPAGPPGPPGPPGPPGPPSG GYD | |
| | GRVGAPGPA | |
| | GRVGAPGPAGA | |
| | GSDGQPGPPGPPGTAGFPGSPGAK | |
| | GSDGSVGPVGPA | |
| | GSDGSVGPVGPAGP | |
| | GSEGPQGVR | |
| | GSEGPQGVRGEPGPPGPA | |
| | GSEGPQGVRGEPGPPGPAGA | |
| | GSPGEAGRPGE | |
| | GSPGEAGRPGEAGLP | |
| | GSPGEAGRPGEAGLPG | |
| | GSPGEAGRPGEAGLPGA | |
| | GSPGEAGRPGEAGLPGAK | |
| | GSPGEAGRPGEAGLPGAKG | |
| | GSPGERGEVGPAGPNG | |
| | GSPGERGEVGPAGPNGFAGPAGAAG QPGAKGE | |
| | GSPGERGEVGPAGPNGFAGPAGAAG QPGAKGER | |
| | GSPGGPGAAGFPGGR | |

| | | |
|---|---|---|
| | GSPGPMGPR | |
| | GSPGPQGPPGSIGPQ | |
| | GSPGRDGSPGAK | |
| | GSPGSPGPDGK | |
| | GSQGSQGPAGPPGPPGPPGPPGPSGG GY | |
| | GSTGEIGPAGPPGPPG | |
| | GTKGPKGENGPVGPT | |
| | GTKGPKGENGPVGPTGPVG | |
| | GTKGPKGENGPVGPTGPVGA | |
| | GTKGPKGENGPVGPTGPVGAA | |
| | GTKGPKGENGPVGPTGPVGAAGPSG PNGPPGPAGSR | |
| | GVAGAPGLPGPR | |
| | GVAGEPGRDGLPGGP | |
| | GVAGEPGRDGLPGGPG | |
| | GVAGEPGRDGLPGGPGL | |
| | GVAGPKGPAGER | |
| | GVDGAPGKDGPR | |
| | GVKGDVGLPGP | |
| | GVPGEDGAPGPMGPR | |
| | GVPGEKGPIG | |
| | GVPGPPGAVGPAGKD | |
| | GVQGPPGPAGPR | |
| | GVVGLPGQRGE | |
| | GVVGLPGQRGER | |
| | GVVGPQGAR | |
| | HGNRGEPGPAGAVGPAGA | |
| | HGNRGEPGPAGAVGPAGAVGPR | |
| | HMWPGDIKA | |
| | HPGPIGPPGPR | |
| | IDGRPGPI | |
| | IGFPGPKGPSGDPGKAGEK | |
| | IGPAGLPGPR | |
| | IGPPGDPGR | |
| | IGPPGPAGAPGDK | |
| | IGPSGPAGK | |
| | IGPSGPAGKD | |
| | IPGIGKPGQDGIPGQPGFPGGK | |
| | IQGPPGPAGE | |
| | IQGPPGPAGEEGK | |
| | IQGPPGPAGEEGKR | |
| | ISGPKGDSGPPGER | |

| | | |
|---|---|---|
| | ISGPPGPPGPAGK | |
| | KAGPPGPK | |
| | KGENGVPGEDGAPGPMGPR | |
| | KGENGVPGENGAPGPMGPR | |
| | KGEPGDKGPR | |
| | KGEPGSSGVDGAPGKDGPR | |
| | LAGPPGESGR | |
| | LDGAKGDAGPA | |
| | LDGAKGDAGPAGPK | |
| | LGAPGPSGAR | |
| | LGPVGNPGPAGPAGPR | |
| | LKGENGVPGEDGAPGPMGPR | |
| | LKGENGVPGENGAPGPMGPR | |
| | LQGLPGTSGPPGENGKPGEPGPK | |
| | LRGIPGSPGGPGSDGKPGPPGSQGETG RPGPPGSPGPR | |
| | LSGPVGPPGNPGANGLPGAK | |
| | LTGSPGSPGPDGK | |
| | LVGEPGPAGSK | |
| | NAGPPGPPGPAGK | |
| | NGDDGEAGKPGRPGE | |
| | NGDDGEAGKPGRPGERGPPGPQG | |
| | NGDDGEAGKPGRPGERGPPGPQGA | |
| | NGEKGETGAPGLKGENGVPGENGAP GPMGPR | |
| | NGETGPQGPPGPTGPSGDKGDTGPPG PQG | |
| | NGVPGEDGAPGPMGPR | |
| | NGVPGENGAPGPMGPR | |
| | PAGLPGPPGER | |
| | PAGPQGPR | |
| | PGAPGPPGKR | |
| | PGAPGTPGER | |
| | PGAVGPLGPR | |
| | PGEAGEPGLPG | |
| | PGEKGPPGDR | |
| | PGGPPGLPGPAGPK | |
| | PGLLGPPGPR | |
| | PGLPGPSGEPGK | |
| | PGLPGPSGEPGKQGPSGA | |
| | PGLPGPSGEPGKQGPSGASGER | |
| | PGPAGPAGPR | |
| | PGPIGPAGAR | |
| | PGPIGPPGPR | |

| | | |
|---|---|---|
| | PGSDGLPGR | |
| | PGSDGLPGRD | |
| | PGSDGLPGRDGAPG | |
| | PPGARGPPGDTGKDGPR | |
| | PPGFDVSGPPGERGSPGIPGAPGPMGPPGSPGLPGK | |
| | PSGPQGPSGPPGPK | |
| | PVGNPGPAGPAGPR | |
| | QGAVGSPGPAGPR | |
| | QGLPGPAGTAGE | |
| | QGLPGTSGPPGENGKPGEPGPK | |
| | QGLPGTSGPPGENGKPGEPGPKGEAGAPGIPGGK | |
| | QGMPGER | |
| | QGPPGPAGEEGK | |
| | QGPPGPPGSPGEQGPSGASGPAGPR | |
| | QGPSGASGERGPPGPMGPPG | |
| | QGVPGDPGAVGPLGPR | |
| | QPGLPGPR | |
| | RGAAGLPGPK | |
| | RGEAGSPGIAGPK | |
| | RGEPGPAGLPGPPGER | |
| | RGETGPAGPAGP | |
| | RGETGPAGPPGAPGAPGAPGPVGPA | |
| | RGETGPAGPSGAPGPAGSR | |
| | RGFPGER | |
| | RGFPGLPGPSGEPGK | |
| | RGIGTPGPKR | |
| | RGIPGSPGGPGSDGKPGPPGSQGETGRPGPPGSPGPR | |
| | RGPAGPQGPR | |
| | RGPLGPPG | |
| | RGPNGDSGRPGEPGLMGPR | |
| | RGPPGAGGPPGPR | |
| | RGPPGESGAAGPTGPIGSR | |
| | RGPPGPPGK | |
| | RGPPGPPGPR | |
| | RGPPGPQGPR | |
| | RGPSGPQGPSGPPGPK | |
| | RGVPGPPGAVGPA | |
| | RGVQGPPGPAGPR | |
| | SAGIPGPFGPR | |
| | SFLPQPPQEK | |
| | SGAAGPTGPIGS | |

| | | |
|---|---|---|
| | SGAAGPTGPIGSR | |
| | SGAPGVPGIAGPR | |
| | SGDRGETGPAGPA | |
| | SGDRGETGPAGPAGPI | |
| | SGDRGETGPAGPAGPIGP | |
| | SGDRGETGPAGPS | |
| | SGDRGETGPAGPSGAPGPA | |
| | SGDRGETGPAGPSGAPGPAGS | |
| | SGDRGETGPAGPSGAPGPAGSR | |
| | SGEPGAPGSKGDTGAK | |
| | SGHPGPIGPPGPR | |
| | SGLDGAKGDAGPAGPK | |
| | SGLDGAKGDAGPAGPKGEPGSPGEN GAPGQMGPR | |
| | SGPAGPRGPPGSAGSPGK | |
| | SGPAGPRGPPGSAGSPGKDGLNGLPG PIGPPGPR | |
| | SGRPGEPGLMGPR | |
| | SPGPQGPPGSIGPQ | |
| | STPVPGLPGPPGPPGR | |
| | TAGFPGSPGAK | |
| | TAGPSGPSGLPGERG | |
| | TGDAGPAGPPGPPGPPGPPGPPSG | |
| | TGDAGPAGPPGPPGPPGPPGPPSGGY D | |
| | TGPPGPAGQDGRPGPPGPPG | |
| | TGPPGPAGQDGRPGPPGPPGA | |
| | TGSPGSPGPDGK | |
| | VAGAPGLPGPR | |
| | VAGEPGRDGLPGGPG | |
| | VAGPKGPAGE | |
| | VAGPKGPAGER | |
| | VAGPPGPSGPPGEK | |
| | VDGAPGKDGPR | |
| | VGAPGPAGAR | |
| | VGEPGPAGSK | |
| | VGEPGPAGSKGESGNKGEPGAVGQP GPPGPSGEEGK | |
| | VGNPGPAGPAGPR | |
| | VGPAGAVGPR | |
| | VGPAGKDGEAGAQ | |
| | VGPPGPPGPAGEK | |
| | VGPPGPSGN | |
| | VGPPGPSGNAGPPGPPGPAGK | |

| | | |
|---|---|---|
| | VGPPGPSGNAGPPGPPGPAGKE | |
| | VGPSGPPGKD | |
| | VGQPGPPGPSGEEGK | |
| | VGSPGPAGPR | |
| | VQGPPGPAGPR | |
| | VQGPPGPAGPRG | |
| | VVGAPGTAGPSGPSGLPGER | |
| | VVGLPGQR | |
| | VVGLPGQRGER | |

**Table S3.** DPPH and FRAP for CH-GL and CH-OPT at times 0, 8, 16 and 24 h for each colonic region.

| | CH-GL | | CH-OPT | |
|---|---|---|---|---|
| **Time (h)** | **DPPH** | **FRAP** | **DPPH** | **FRAP** |
| | | **Ascending colon** | | |
| 0 | 24.00±6.05 | 440.82±270.11 | 17.53±0.68 | 273.39±2.49 |
| 8 | 24.05±2.05 | 392.93±92.98 | 18.63±2.03 | 315.14±56.17 |
| 16 | 27.68±2.63 | 454.30±50.21 | 28.25±0.85* | 370.81±43.24 |
| 24 | 29.10±1.20 | 424.08±28.36 | 26.88±1.28* | 390.69±73.07 |
| | | **Transverse colon** | | |
| 0 | 20.65±8.75 | 431.06±247.33 | 15.40±0.45 | 236.11±55.67 |
| 8 | 21.20±3.85 | 392.00±40.91 | 21.58±3.93 | 255.00±31.81 |
| 16 | 24.08±0.43 | 328.78±74.38 | 16.13±1.28 | 297.74±1.99 |
| 24 | 17.55±4.25 | 292.52±66.95 | 23.73±0.03 | 323.09±55.17 |
| | | **Descending colon** | | |
| 0 | 29.35±9.85 | 520.32±304.04 | 22.50±1.10 | 245.05±26.84 |
| 8 | 24.25±11.45 | 699.77±99.49 | 19.98±0.43 | 170.49±39.77 |
| 16 | 28.28±9.43 | 309.25±110.65 | 21.15±0.50 | 247.54±42.25 |
| 24 | 20.68±3.73 | 492.42±71.59 | 19.55±0.01 | 278.36±23.36 |

Values are expressed as mean ± SEM in mM. DPPH is reported in mM Trolox Eq. FRAP is reported in µM ascorbic acid equivalents. Within a column, * symbol indicates significant differences from control (Time 0 h) ($p<0.05$).
